# Encapsulation of Bacteriophages in Alginate Beads: Improved Viability Under Harsh Simulated Gastric and Intestinal Conditions for Phage Therapy Applications

**DOI:** 10.3390/ph19030363

**Published:** 2026-02-25

**Authors:** Sally Ameen Almekhlafi, Mohamed A. Farrag, Mona S. Al-Wahibi, Sarah Al-Rashed, Basmah Mohammed Almaarik, Najat A. Y. Marraiki

**Affiliations:** 1 Department of Botany and Microbiology, College of Science, King Saud University, Riyadh 11451, Saudi Arabia; 442204462@student.ksu.edu.sa (S.A.A.); malwhibi@ksu.edu.sa (M.S.A.-W.); salrashed@ksu.edu.sa (S.A.-R.); 2 Clinical Laboratory Sciences, King Saud University, Riyadh 11451, Saudi Arabia; balmaarik@ksu.edu.sa; 3 Microbiology Unit, Central Research Laboratory (CRL), King Saud University, Riyadh 11451, Saudi Arabia

**Keywords:** bacteriophage therapy, phage encapsulation, alginate beads, simulated gastrointestinal conditions, oral phage delivery, encapsulation efficiency, phage viability

## Abstract

**Background/Objectives**: Bacteriophages offer a promising alternative to conventional antibiotics. However, their therapeutic efficacy is often limited by instability in harsh environmental conditions, particularly within the gastrointestinal tract. This study aimed to isolate lytic bacteriophages from wastewater and evaluate the protective capacity of sodium alginate encapsulation against various stressors to enable effective oral delivery. **Methods**: Four distinct lytic phages (As, Ec, Pa, Gc) were isolated from wastewater and characterized by Transmission Electron Microscopy (TEM) and PCR, confirming their families (Siphoviridae, Podoviridae, Myoviridae). These phages demonstrated potent lytic activity against diverse bacterial pathogens, including *Aeromonas hydrophila*, *Escherichia coli*, *Pseudomonas aeruginosa*, and *Glutamicbacter creatinolyticus*. The phages were encapsulated in 5% sodium alginate via an extrusion method. Stability was assessed under extreme pH (2.0 and 13), at elevated temperature (up to 80 °C), and in simulated gastrointestinal transit. **Results**: Encapsulation efficiency exceeded 95%. Unencapsulated phages were completely inactivated at pH 2.0 within 10 min, whereas encapsulated phages maintained significant viability (3.06–3.43 log PFU/mL). Encapsulation also significantly enhanced phage survival under extreme alkaline conditions and elevated temperatures. In simulated gastrointestinal transit, encapsulated phages exhibited superior recovery (2.50 log PFU/mL) compared to their free counterparts (≤1 log PFU/mL). Long-term storage evaluations over three months further confirmed the robust stability of the encapsulated formulations at both 4 °C and 21 °C. **Conclusions**: Sodium alginate encapsulation effectively shields bacteriophages from severe environmental degradation, particularly acidic gastric stress, enhancing their potential for oral delivery. These findings support the development of stable, formulated phage products for diverse practical applications in phage therapy to combat antimicrobial resistance.

## 1. Introduction

The global crisis of the antimicrobial resistance (AMR) phenomenon is escalating. Traditional antibiotics are increasingly ineffective against multidrug-resistant (MDR) bacteria. This results in millions of deaths annually, driving an urgent need for innovative therapeutic alternatives [1,2,3]. Bacteriophages, or phages, are viruses that specifically infect and lyse targeted bacteria. They represent a precise and promising approach for combating infections, particularly those caused by pathogenic bacteria such as *E. coli*, *P. aeruginosa*, and *A. hydrophila* [4,5,6,7]. Wastewater treatment plants serve as rich environmental reservoirs for isolating diverse lytic phages. These sites harbor complex microbial communities, including MDR strains, enabling the discovery of novel phages with broad therapeutic potential [8,9,10,11,12]. Recent studies have successfully isolated phages from municipal wastewater, demonstrating strong lytic activity against Gram-negative pathogens. This underscores their role in addressing AMR in both environmental and clinical settings [10,13,14,15]. Phage therapy offers several advantages, including specificity, minimal impact on the host microbiome, and the ability to co-evolve with bacterial hosts. These characteristics make it especially suitable for gastrointestinal infections where targeted bacterial clearance is critical [16,17].

Despite the therapeutic promise of bacteriophages, their oral application faces a significant challenge. Phages are inherently vulnerable to the harsh conditions of the gastrointestinal tract (GIT). The highly acidic gastric environment (pH 1.5–3.5), coupled with proteolytic enzymes like pepsin, rapidly inactivates free phages. Subsequent exposure to bile salts and pancreatic enzymes in the intestine further contributes to this degradation. Phage viability often reduces to undetectable levels within minutes to hours [18,19,20,21,22]. This instability severely limits the delivery of sufficient viable phages to the infection site in the intestines, thereby diminishing therapeutic efficacy. Protective strategies are necessary to ensure phage survival during gastric transit and controlled release in the more neutral intestinal milieu [22,23]. Without such protection, oral phage administration fails to achieve adequate bioavailability. This highlights the need for advanced formulation approaches that can overcome these physiological barriers and enhance phage-based therapy for gut-related MDR infections [17,18]. Recent investigations confirm that free phages experience near-total inactivation at pH < 3 after short exposure, emphasizing the critical role of encapsulation in preserving lytic activity [22,23].

Encapsulation using sodium alginate has emerged as a highly effective pharmaceutical strategy. Alginate is a biocompatible, biodegradable, and naturally derived polysaccharide. It safeguards bacteriophages during gastrointestinal passage while enabling pH-responsive release in the intestines [18,22]. Alginate forms stable, acid-resistant gels in the presence of calcium ions, shielding encapsulated phages from low gastric pH and enzymatic degradation. It subsequently swells and disintegrates at higher intestinal pH values (approximately 6.8–7.5), facilitating targeted phage liberation [22,24,25]. Recent advancements show that alginate-based formulations, sometimes combined with buffering agents or additional polymers like carrageenan, achieve high encapsulation efficiencies. They maintain high phage viability after prolonged exposure to simulated gastric fluid (SGF) and promote efficient release in simulated intestinal fluid (SIF) with sustained lytic activity [18,22]. These properties position alginate encapsulation as a scalable, cost-effective, and safe method for oral phage delivery, with proven potential to improve therapeutic outcomes in animal models and pave the way for human applications [22,23]. Moreover, incorporation of CaCO_3_ or other stabilizers further enhances protection against extreme acidity, making this approach ideal for diverse phage morphologies [18,22].

The present study addresses critical gaps in phage therapy research, particularly within Saudi Arabia. While bacteriophage isolation from Saudi wastewater has been reported previously [26,27], and alginate-based encapsulation of phages for gastrointestinal protection has been explored globally, this work offers a novel integration. It combines lytic phages isolated from wastewater of Riyadh’s arid-environment treatment plants with a tailored 5% alginate bead formulation optimized for high encapsulation efficiency (>95%) and robust in vitro gastrointestinal stability. Given the extreme high-temperature, low-humidity, and often high-salinity conditions characteristic of Riyadh’s wastewater environments, these phages may have evolved greater intrinsic structural robustness compared to those isolated in temperate climates, a property that could further enhance their suitability for protected oral delivery formulations. This study therefore establishes a strong pharmaceutical platform for improving the oral delivery, bioavailability, and therapeutic efficacy of bacteriophages targeting intestinal infections. This approach is particularly relevant to regions like Saudi Arabia, which have limited prior phage formulation research and a scattered phage research landscape. By evaluating encapsulation efficiency, particle morphology, and the comparative stability of free versus encapsulated phages in simulated gastrointestinal environments, this research contributes significantly to the global effort against AMR through sustainable, targeted antimicrobial strategies.

## 2. Results

### 2.1. Isolation and Identification of Bacteria from Wastewater

A comprehensive analysis of wastewater samples yielded a total of 48 bacterial isolates. From these, 10 distinct bacterial strains were definitively identified using the VITEK-2 system. This identified cohort represented a diverse microbiological landscape, encompassing both Gram-positive and Gram-negative bacteria, with varying morphologies including cocci and bacilli. Notably, several of these identified isolates are recognized as clinically relevant human pathogens, such as *A. hydrophila*, *E. coli*, *P. aeruginosa*, and *G. creatinolyticus*. These strains show varied phenotypes and pathogenic potential, indicating many suitable bacterial hosts for phage isolation in wastewater. This diversity provides a robust foundation for investigating phage-host interactions and potential therapeutic applications.

### 2.2. Isolation and Morphological Characterization of Bacteriophages

**Figure 1:**
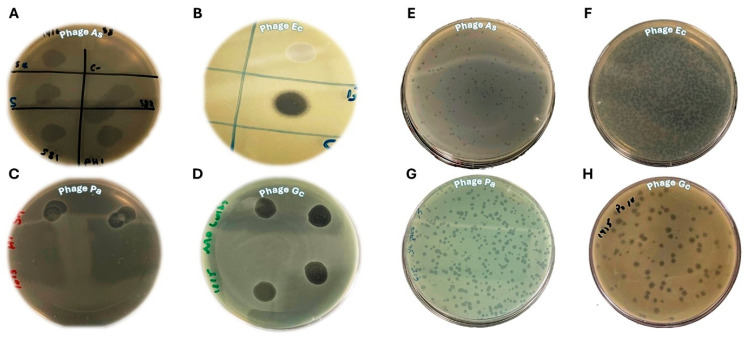
Lytic activity and plaque morphology of bacteriophages. The lytic activity of four bacteriophages (Phage As, Phage Ec, Phage Pa, and Phage Gc) was evaluated using spot assays (**A**–**D**) and plaque assays (**E**–**H**). (**A**–**D**) Spot Assays: clear zones of inhibition indicate the lytic activity of Phage As (**A**), Phage Ec (**B**), Phage Pa (**C**), and Phage Gc (**D**) against their respective bacterial hosts. (**E**–**H**) Plaque Assays: Representative images showing plaque formation on bacterial lawns. Phage As (**E**), Phage Ec (**F**), Phage Pa (**G**) Phage Gc (**H**). These assays collectively demonstrate the lytic potential and diverse plaque morphologies of the characterized bacteriophages.

Four phages were isolated, and their lytic capabilities were subsequently assessed using both spot assay and plaque assay methods (Figure 1). Spot assays (Figure 1A–D) qualitatively demonstrated the lytic activity of all four phages against their respective bacterial hosts, evidenced by clear zones of inhibition at the points of phage application. Phage As (Figure 1A) produced multiple distinct, clear spots, while Phage Ec (Figure 1B) exhibited a prominent, well-defined clear zone. Phage Pa (Figure 1C) showed two clear zones of lysis, and Phage Gc (Figure 1D) displayed four distinct and robust zones of lysis. Further quantitative analysis through plaque assays (Figure 1E–H) revealed variations in plaque size, clarity, and number. Phage As (Figure 1E) formed numerous small, somewhat turbid plaques, suggesting either incomplete lysis or the presence of lysogenic phages. Phage Ec (Figure 1F) produced a high density of very small, almost confluent plaques, indicative of highly efficient lytic activity and a high titer. Phage Pa (Figure 1G) generated numerous distinct, clear plaques of varying sizes, signifying efficient host lysis. Lastly, Phage Gc (Figure 1H) formed distinct, relatively larger, and clear plaques, though appearing in lower numbers compared to Phage Ec and Phage Pa. Additional images of the plaque assays of the four phages are provided in the Supplementary Materials (Figures S1–S7). These combined morphological and lytic characterizations provide foundational insights into the diversity and functional properties of these bacteriophages.

**Figure 2:**
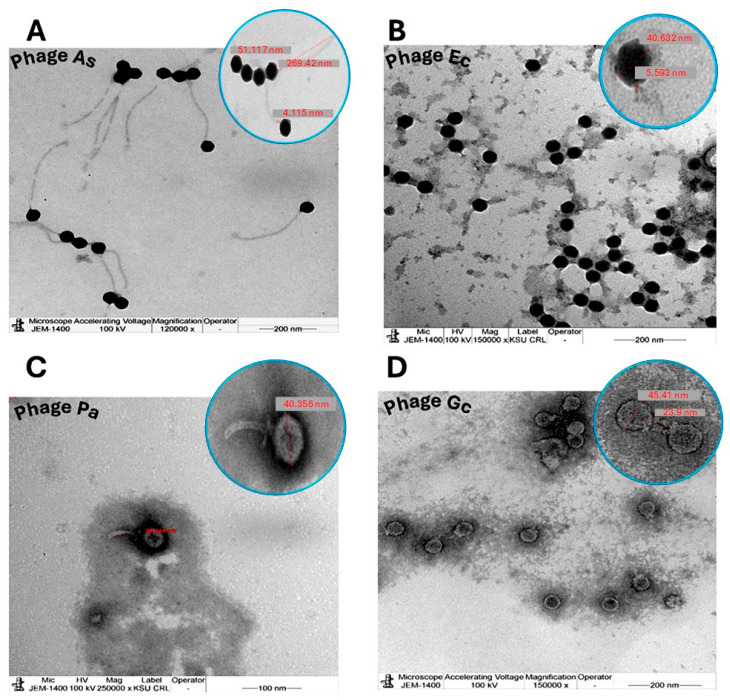
Morphological characterization of bacteriophages via transmission electron microscopy (TEM). Micrographs obtained using a JEM-1400 TEM (JEOL Ltd., Akishima, Tokyo, Japan) at 100 kV illustrate the structural diversity of four distinct phage isolates: (**A**) Phage As, (**B**) Phage Ec, (**C**) Phage Pa, and (**D**) Phage Gc. Circular insets provide high-magnification views with representative dimensions (in nanometers) for head diameter and tail length. Scale bars represent 200 nm (**A**,**B**,**D**) and 100 nm (**C**).

TEM was utilized to characterize the morphology of the four phages (Figure 2). Phage As (Figure 2A) exhibited a siphoviral morphology, characterized by an isometric head with an approximate diameter of 51.117 nm and a long, flexible, non-contractile tail extending to 269.423 nm. Phage Ec (Figure 2B) displayed a podoviral morphology, featuring a prominent, roughly spherical, electron-dense head measuring approximately 40.632 nm in diameter, with a very short, almost imperceptible tail. Phage Pa (Figure 2C) presented an oval-shaped head with a discernible short, slightly curved tail. The magnified inset indicated a head length of approximately 40.356 nm along its major axis (major axis) and a short, slightly curved tail. Phage Gc (Figure 2D) showed icosahedral heads that appeared relatively lighter and more translucent, with an average diameter of approximately 45.41 nm. Additional TEM images demonstrating the morphological features of the four isolated phages, such as icosahedral heads and contractile or non-contractile tails consistent with standard bacteriophage classification, are presented in the Supplementary Materials (Figures S8–S18).

### 2.3. Molecular Identification of Bacteriophages

To corroborate the TEM-based classifications, PCR amplification was performed using family-specific primers. For Phage As, PCR using the Siph primer set yielded a clear amplicon of 459 bp, confirming its affiliation with the Siphoviridae family. Phage Pa produced amplicons with both G23 (568 bp) and G43 (476 bp) primer sets, indicative of Myoviridae membership. The dual amplification suggests potential variability in the targeted regions but aligns with the contractile tail observed in TEM, reinforcing its classification. Phage Ec amplified a 774 bp product exclusively with the Pod primer set, consistent with its Podoviridae designation from TEM imaging. Similarly, Phage Gc showed specific amplification of the 774 bp fragment using the Pod primers, matching its morphological features and confirming Podoviridae classification. No cross-amplification was detected with Myoviridae or Siphoviridae primers.

### 2.4. Host Range and Lytic Efficiency

**Figure 3:**
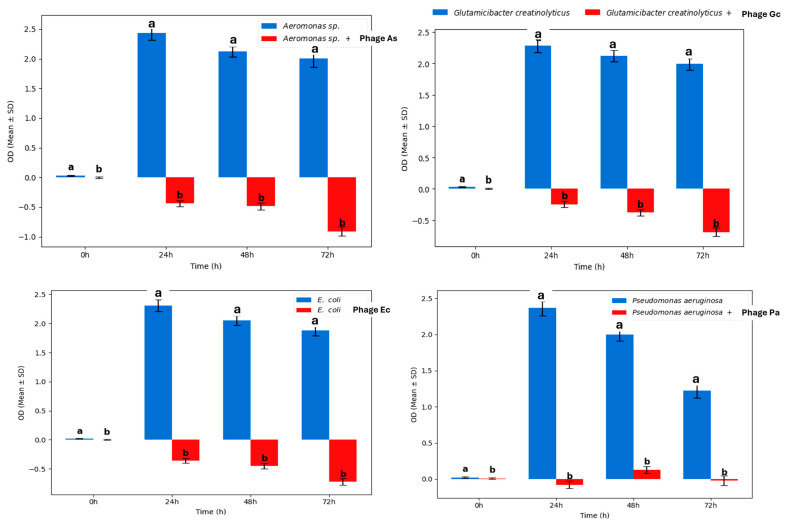
In vitro lytic activity of bacteriophages against their respective bacterial hosts. Optical density (OD_600_) was monitored over 72 h for *Aeromonas* sp. (Phage As), *G. creatinolyticus* (Phage Gc), *E. coli* (Phage Ec), and *P. aeruginosa* (Phage Pa) in the absence (blue bars) or presence (red bars) of phages. Data are presented as mean ± SD (*n* = 3). Different letters (a, b) indicate statistically significant differences (*p* < 0.05) at each time point.

The isolated bacteriophages demonstrated diverse host specificities when tested against a panel of 50 bacterial strains. Phages As and Ec exhibited relatively narrow host ranges, with phage Ec specifically lysing *E. coli* and *Kocuria rhizophila*, and phage As targeting *Aeromonas hydrophila* and *Bacillus cereus*. In contrast, phages Gc and Pa displayed broader lytic activity, effectively targeting multiple bacterial genera including *P. aeruginosa* and *G. creatinolyticus*. The lytic efficacy of four bacteriophages (Phage As, Phage Gc, Phage Ec, and Phage Pa) was evaluated against their respective host bacteria (*Aeromonas* sp., *G. creatinolyticus*, *E. coli*, and *P. aeruginosa*) over 72 h using optical density (OD_600_) measurements. In the absence of phages, all bacterial strains exhibited robust growth, reaching OD values of approximately 2.0–2.5 within 24 h and maintaining high densities thereafter. In contrast, phage-treated cultures displayed rapid and significant inhibition, with OD values dropping to near zero or negative levels by 24 h and remaining suppressed throughout the 72 h incubation period (*p* < 0.05 compared to controls at all time points beyond 0 h) (Figure 3). These results demonstrate the potent, sustained lytic activity of the tested phages against taxonomically diverse bacterial pathogens under the experimental conditions. These findings highlight the varied lytic capabilities and host specificities among the isolated phages, which are crucial considerations for their potential application in phage therapy.

### 2.5. Encapsulation Efficiency

To ensure the effective delivery of bacteriophages As, Gc, Ec, and Pa through challenging environments such as the gastrointestinal tract, encapsulation was employed. The primary objective was to protect the phages from harsh conditions, achieve high encapsulation efficiency (EE%), and facilitate controlled release. The encapsulation process involved formulations designed to provide robust protection. All four phages demonstrated high encapsulation efficiencies, consistently exceeding 95% across all formulations tested. This high efficiency was further corroborated by the minimal recovery of free phages in the crosslinking solution (less than 0.5% of the total amount), indicating successful phage entrapment within the capsules.

### 2.6. Comparative Stability of Phages Under Physical Stress

#### 2.6.1. pH Conditions (Simulated Gastric and Gastrointestinal Environments)

The viability of both unencapsulated and encapsulated phages (As, Gc, Ec, and Pa) was assessed following incubation in various pH conditions, simulating the gastric and intestinal environments. Exposure to highly acidic conditions (pH 2.0 and pH 2.5) for 10 min resulted in a significant reduction in unencapsulated phage titres, often falling to 1 log PFU/mL or below detectable levels (<1 log PFU/mL) from an initial inoculum of approximately 6 log PFU/mL. In contrast, encapsulation significantly enhanced phage survival. At pH 2.0, after 10 min, encapsulated phages maintained detectable titres, ranging from 3.06 log PFU/mL (phage As) to 3.43 log PFU/mL (phage Ec). The protective effect was even more pronounced at pH 2.5, where encapsulated phages maintained titres between 3.30 and 4.00 log PFU/mL, significantly higher than the ≤1 log PFU/mL observed for unencapsulated phages (*p* < 0.001) (Table 1).

The stability of phages was also evaluated under alkaline conditions (pH 10 and pH 13). At pH 10, unencapsulated phages showed moderate stability, with titres ranging from 2.12 log PFU/mL (phage As) to 2.37 log PFU/mL (phage Ec). Encapsulation provided a protective boost, increasing recovery to 3.15 log PFU/mL for phage As and 3.49 log PFU/mL for phage Ec and 3.40 log PFU/mL for phage Gc. At the extreme alkaline pH of 13, the unencapsulated phages were highly susceptible, with Gc and Pa falling to 1.00 log PFU/mL, while phages As and Ec dropped to 1.18 log and 1.50 log PFU/mL, respectively. The four encapsulated phages showed significantly better resilience at pH 13, with titres reaching 2.28 log PFU/mL for phage As and 2.63 log PFU/mL for phage Ec.

Extending the incubation to 60 min at pH 2.0 led to complete inactivation for the unencapsulated phages (As, Gc, Ec, and Pa) (≤1 log PFU/mL), while encapsulated phages maintained a titre of 2.20 log PFU/mL. At pH 3.0, both forms showed high stability, with unencapsulated phages at 5.50 log PFU/mL and encapsulated phages at 6.00 log PFU/mL. Recovery at the control pH of 7.5 remained unchanged at 6.00 log PFU/mL for both forms. Under sequential simulated gastrointestinal conditions, unencapsulated phages were reduced to 1.00 log PFU/mL after SGF exposure, whereas encapsulated phages exhibited significant recovery at 2.50 log PFU/mL, demonstrating the critical role of encapsulation in protecting phages during gastric transit (Table 1).

#### 2.6.2. Temperature Variations

The stability of phages was evaluated across a range of temperatures. At elevated temperatures, encapsulation significantly enhanced survival. After 30 min at 50 °C, unencapsulated phages experienced significant reductions, with titres ranging from 1.39 log PFU/mL (Pa) to 2.35 log PFU/mL (As). Encapsulated phages showed improved survival, with titres reaching 3.35 log PFU/mL for phage As and 2.87 log PFU/mL for phage Gc. At 70 °C, unencapsulated phages underwent rapid inactivation (1.00 log PFU/mL), while encapsulated phages maintained higher viability, particularly phage Ec at 2.74 log PFU/mL and others at 1.50 log PFU/mL. Even at 80 °C, while both forms faced rapid inactivation, encapsulated phages still showed a detectable titre of 2.52 log PFU/mL compared to 1.52 log PFU/mL for unencapsulated forms. Conversely, at refrigeration (4–5 °C) and ambient (21 °C) temperatures, both forms maintained high viability at 6.00 log PFU/mL. Freezing at −80 °C also showed good stability, with unencapsulated phages at 5.50 log PFU/mL and encapsulated phages at 6.00 log PFU/mL (Table 1).

#### 2.6.3. Storage Duration and Conditions

Encapsulation significantly extended the long-term viability of phages over 3 months. In dry and humid conditions at 21 °C, both forms maintained high viability at 6.00 log PFU/mL, indicating excellent stability in the tested formulations. Encapsulation significantly extended the long-term viability of phages under wet storage conditions at 4 °C over 3 months (5.20 log PFU/mL vs. 4.80 log PFU/mL for free phages; *p* < 0.05), whereas both free and encapsulated phages retained high and comparable viability (~6.00 log PFU/mL) under dry and humid conditions at 21 °C (Table 1). Phages Ec and Gc (both Podoviridae) showed the most consistent stability when encapsulated, likely due to their compact morphology with short, non-contractile tails that confer greater inherent resilience compared to longer-tailed Siphoviridae and Myoviridae.

### 2.7. Morphology of the Phage Alginate Capsules

**Figure 4:**
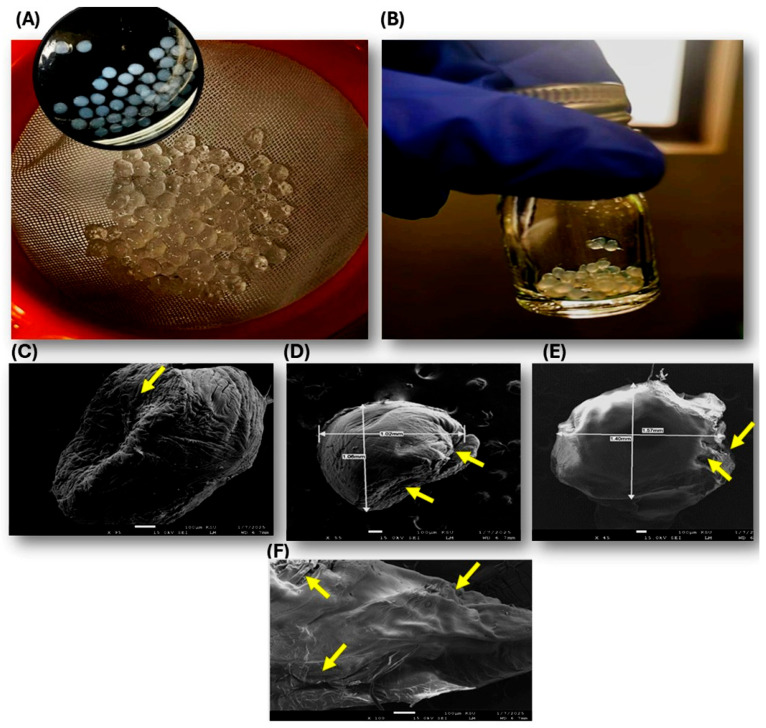
Morphology of phage alginate capsules. (**A**) Macroscopic view of wet capsules on a sieve (inset: close-up of spherical beads). (**B**) Wet capsules in a glass vial held in a gloved hand. (**C**,**D**) SEM images of dry alginate capsules. The capsules display a compact, wrinkled, and highly textured surface with characteristic folding and cracking patterns resulting from dehydration of the hydrogel structure. Scale bar: 100 µm (left: ×55, right: ×95), (**E**,**F**) SEM images of hydrated (wet) alginate capsules. The capsules exhibit a soft, irregular, and highly hydrated surface morphology with a wrinkled and folded outer layer typical of swollen hydrogel beads. Scale bar: 100 µm (left: ×45, right: ×100). Yellow arrows refer to wrinkled irregular surfaces appearing on both dry and wet capsules.

The morphology and size distribution of the phage capsules were characterized using optical photography and field emission scanning electron microscopy (SEM). Macroscopic examination revealed that the wet capsules appeared as translucent, spherical to slightly oval gel beads with a uniform size distribution (Figure 4A, inset). When collected on a sieve, the beads exhibited a consistent diameter of approximately 1–2 mm, displaying a hydrated, glossy appearance typical of alginate-based hydrogels (Figure 4A). In a laboratory vial, the wet capsules maintained their structural integrity and spherical shape, with no visible aggregation or deformation (Figure 4B). In Supplementary Materials, Figures S19–S24 show several images of phage capsules captured by digital camera. SEM analysis at low magnifications confirmed the spherical geometry of individual capsules, with diameters ranging from approximately 1.02 mm to 1.57 mm (Figure 4C–E). The surface morphology was characterized by a rough, wrinkled, and irregular texture, featuring pronounced folds and crevices (Figure 4C). Higher-resolution imaging highlighted a heterogeneous external surface with microporous features and occasional protrusions (Figure 4D). Some particles displayed partial collapse or membrane-like wrinkling, likely attributable to dehydration during sample preparation. These observations are consistent with typical alginate capsules formed by extrusion or emulsification methods, where surface irregularities arise from rapid gelation and drying processes. Hydrated alginate beads displayed a similar wrinkled appearance (Figure 4E,F). Additional SEM images of both wet and dried capsules are shown in Supplementary Materials (Figures S25–S30). Overall, the alginate capsules exhibited good structural uniformity at the macroscopic level and a characteristic wrinkled morphology at the microscale, supporting their suitability for encapsulating bacteriophages while facilitating controlled release under gastrointestinal conditions.

## 3. Discussion

This study successfully isolated and characterized four novel bacteriophages (As, Ec, Pa, Gc) from municipal wastewater, targeting clinically relevant pathogens: *A. hydrophila*, *E. coli*, *P. aeruginosa*, and *G. creatinolyticus*. Wastewater treatment plants (WWTPs) are rich reservoirs for diverse viral populations, modulating bacterial communities and serving as therapeutic agent sources [12,28,29,30]. These environments encourage phage proliferation due to high bacterial host density and diversity, facilitating frequent phage-host interactions and novel lytic capabilities, making them prime for bioprospecting [11,31,32,33]. The identification of *G. creatinolyticus* as a host is notable, as this species is increasingly recognized for its pathogenic potential in humans and animals, linked to opportunistic infections, bacteremia, and abscesses [34,35,36]. While previous studies focused on *Glutamicibacter* phages in food systems [37], this study provides critical evidence of their presence and lytic activity in environmental wastewater, expanding their known ecological niche and suggesting a significant role in wastewater microbial dynamics.

Morphological analysis via TEM and molecular classification using family-specific primers revealed a diverse viral landscape reflecting the taxonomic breadth of the Caudoviricetes class. Phage As was identified as Siphoviridae (long, non-contractile tail, isometric head), Phage Pa as Myoviridae (robust, contractile tail), and Phage Ec as Podoviridae (short, non-contractile tail). These findings align with recent metagenomic surveys of urban wastewater, consistently identifying these three families as dominant virome components, often exceeding 90% of identifiable viral sequences [12,38]. PCR-based markers (G23, G43, Siph, Pod) for family-level identification offer a robust, cost-effective, and rapid alternative to whole-genome sequencing (WGS) for preliminary phage classification. While WGS remains the gold standard for detailed genomic characterization, PCR-based screening is highly relevant in resource-limited settings and for high-throughput environmental monitoring [39,40]. The correlation between our TEM observations and PCR results validates these primer sets for environmental phage surveillance.

A critical challenge in oral phage therapy is the extreme acidity of the gastric environment, leading to rapid and irreversible inactivation of free phage particles. Our results demonstrated a stark contrast: free phages were almost entirely inactivated within 10–60 min at pH 2.0 and 2.5, while alginate-encapsulated phages maintained significant viability (3.06–4.00 log PFU/mL). This protective effect is primarily attributed to the dense, cross-linked calcium-alginate matrix. The higher 5% alginate concentration was chosen to maximize acid resistance; this results in somewhat slower release at intestinal pH compared to 2–3% formulations, but still enables effective phage recovery (~2.50 log PFU/mL) in the simulated GI model. At low pH, alginate polymer transitions to an insoluble alginic acid gel, significantly reducing hydrogen ion diffusion into the microcapsule core, shielding internal phages from denaturing conditions [18,41]. Similar enhancements in gastrointestinal stability have been reported for *Salmonella* phages, where alginate-based systems provided a buffered microenvironment preserving lytic activity during simulated gastric transit [42,43,44]. The significantly higher survival rates (*p* < 0.001) underscore the necessity of advanced formulation strategies for effective oral delivery.

Exposure to extreme alkaline pH (13) led to substantial inactivation of free phages, most likely due to capsid protein denaturation and tail fiber disruption [45]. The partial protection afforded by encapsulation at this pH is consistent with limited but non-zero diffusion of hydroxyl ions through the alginate matrix, together with some polymer hydrolysis under such extreme conditions [46,47].

The calcium-alginate beads are designed to provide strong protection in the acidic gastric environment (pH 2.0–3.5), while swelling and gradual disintegration occur in the neutral-to-alkaline small intestine (pH ≥ 6.0–7.5), where bile salts, phosphates, and other anions facilitate disruption of the Ca^2+^ crosslinks. This pH-responsive behavior promotes release of viable phages in the intestinal compartment, where target bacterial pathogens are primarily located. The wrinkled surface morphology observed by SEM likely contributes to increased surface area and facilitates more rapid hydration and erosion once the beads reach the higher pH of the small intestine [18,19,22].

Furthermore, the study comprehensively evaluated the stability of both free and encapsulated phages across a wide range of temperatures and storage conditions. The protective benefit of encapsulation on long-term storage was most evident under wet refrigerated conditions (4 °C), whereas under dry and humid ambient conditions (21 °C) both formulations demonstrated excellent and comparable stability (~6.00 log PFU/mL) over 3 months. This enhanced long-term stability is likely due to phage immobilization within the polymer network, restricting molecular motion and protecting viral capsids against dehydration-induced structural damage and oxidative stress [41]. In contrast, free phages are highly susceptible to titer loss, often requiring continuous refrigeration or complex lyophilization [21,48]. However, in our study, titer loss of free phages was not detected under these conditions. The robust performance of our alginate capsules at ambient temperatures suggests seamless integration into animal feed or environmental decontamination protocols without stringent cold-chain logistics, particularly relevant for large-scale farming operations. Tests at elevated temperatures (50–80 °C) were included as accelerated stress conditions to assess the thermal resilience of the encapsulated phages beyond physiological or storage-relevant ranges. These conditions are not clinically relevant but provide a stringent benchmark for formulation robustness [19,49,50].

The lytic efficacy of the isolated phages against their respective hosts was rigorously demonstrated through qualitative spot assays and quantitative growth inhibition curves over 72 h. OD_600_ measurements revealed that all four phages significantly suppressed host growth, with Phage Ec and Phage Pa showing rapid and sustained lytic activity. Phages Gc and Pa exhibited broader host ranges across the 50 bacterial strains tested, suggesting utility in “cocktail” formulations targeting multiple strains or even different genera simultaneously. Broad-spectrum lytic activity is a highly sought-after trait in phage therapy, reducing the need for precise host identification and providing resilient defense against phage-resistant bacterial mutants [17,41]. Variations in plaque morphology—from small, turbid (Phage As) to large, clear, well-defined (Phage Gc)—provide insights into phage life cycles and infection kinetics. Turbid plaques often indicate temperate phages or incomplete lysis, while clear plaques signal strictly lytic (virulent) phages, preferred for therapeutic applications to avoid horizontal gene transfer risks associated with lysogeny [51,52].

The particularly robust stability observed for encapsulated Phages Ec and Gc (both classified as Podoviridae) across multiple stress conditions, including long-term storage and exposure to extreme pH and temperature, may be related to their compact virion morphology. Compared to Siphoviridae (long, flexible non-contractile tails) and Myoviridae (long contractile tails), podoviruses typically possess smaller isometric heads and very short or almost absent tails, resulting in a structurally simpler and more mechanically resistant architecture [53,54]. This compactness is considered to confer greater inherent tolerance to physical stressors, conformational damage, and chemical insults, which likely become more pronounced when the phages are immobilized and protected within the calcium-alginate matrix [19,55]. These structural features provide a plausible explanation for the consistently higher viability retention of the two podoviruses under the tested encapsulated conditions.

While this study successfully isolated and characterized lytic bacteriophages targeting four clinically relevant pathogens, the primary objective was to demonstrate the protective efficacy of 5% sodium alginate encapsulation against harsh simulated gastric (pH 2.0–2.5) and intestinal conditions, enabling effective oral phage delivery. The encapsulation system achieved high survival (>3 log PFU/mL at pH 2.0 after 10 min) and significant recovery after sequential simulated gastric and intestinal fluid exposure, providing comparable protection across all four morphologically diverse phages. These four pathogens occupy distinct clinical and ecological niches, with *A. hydrophila* and diarrheagenic *E. coli* most commonly associated with gastrointestinal infections [56,57], while *P. aeruginosa* frequently colonizes the gut in high-risk hospitalized patients, where oral decolonization may help reduce the risk of systemic dissemination [58,59]. In contrast, *G. creatinolyticus* is more often linked to extraintestinal opportunistic infections such as bacteremia and abscesses [36]. Given these differences in infection sites and pathogenic behavior, a detailed comparative analysis of their action modes, including biofilm formation, MDR profiles, and precise infection locations, was beyond the scope of the present study. It is nevertheless well established that these pathogens differ markedly in pathogenic behavior and resistance patterns [60,61]. Consequently, while oral administration is most appropriate for enteric infections and gut carriage reduction, infections at respiratory, wound, or other extraintestinal sites would likely require alternative delivery routes (topical, nebulized, or intravenous) to achieve therapeutic efficacy [17,62]. The robust, pH-responsive alginate platform developed here represents a crucial foundational step toward stable oral phage formulations and can serve as a versatile delivery system for future pathogen-specific optimizations and cocktail development.

Though the findings of this study are promising, limitations must be acknowledged. Firstly, the molecular classification relied on family-specific PCR primers, which, while cost-effective, do not provide the detailed genomic information necessary to rule out the presence of undesirable genes, such as virulence or lysogeny-associated genes. Future work should prioritize Whole-Genome Sequencing of the four lytic phages to confirm their strictly lytic nature and ensure their safety for therapeutic application. Secondly, the in vitro stability assays, while rigorous, only simulate the harsh conditions of the gastrointestinal tract. In vivo studies using animal models are essential to validate the protective efficacy of the alginate capsules against the complex physiological environment of the gut, including the effects of bile salts and digestive enzymes. Thirdly, although total recoverable viable phages were quantified after complete bead disintegration following simulated gastrointestinal transit, time-resolved release kinetics in simulated intestinal fluid were not assessed in this proof-of-concept study. Finally, a more comprehensive host range analysis against a larger, geographically diverse collection of clinical isolates is required to confirm the broad-spectrum potential of the phages, particularly Phages Gc and Pa, for use in a therapeutic cocktail.

## 4. Materials and Methods

### 4.1. Sample Collection and Processing

Raw wastewater samples were collected from two wastewater treatment plants, KSU-WWTP (24°43′33.8″ N, 46°36′27.9″ E) and MN-WWTP (24°35′12.0″ N, 46°43′53.0″ E), over a two-year period (2022–2024). Safety precautions were considered during collection and processing of samples. Samples were allowed to settle for one hour at room temperature to remove solid particles. Each sample was then divided into three 300 mL aliquots and stored in sterilized glass containers.

### 4.2. Bacterial Isolation and Identification

Bacterial strains were isolated by direct plating of diluted wastewater samples onto nutrient agar (NA) medium (1% peptone, 4% dextrose, 3% agar, distilled water, pH 7.0). Plates were incubated at 37 °C for 24 h. Individual colonies were purified by subculturing twice using the streak plate method. A total of 48 bacterial strains were isolated and maintained on nutrient agar for further analysis. Purified isolates were categorized as Gram-negative or Gram-positive using the Gram staining protocol. Bacterial suspensions were prepared for each isolate by emulsifying them in 0.45% saline to a 0.5 McFarland turbidity standard. Gram-negative bacteria were identified using the VITEK^®®^2 GN ID card (BioMérieux, Marcy-l’Étoile, France), and Gram-positive isolates were identified using the VITEK^®®^2 GP card. Bacterial strains were stored at −20 °C in nutrient broth containing 25% (*v*/*v*) glycerol in sterile screw-capped Eppendorf tubes.

### 4.3. Bacteriophage Isolation and Assays

Bacteriophage isolation was performed using a modified enrichment method. Wastewater samples were centrifuged at 5000 rpm for 25 min, and the supernatant was filtered through a sterile 0.45 µm membrane filter. Ten milliliters of the filtered supernatant was transferred to a Falcon tube containing 2 mL of 1× nutrient broth and 100 µL of a log-phase target bacterial culture (OD_600_ of 0.4). The mixture was incubated at 37 °C with shaking (120 rpm) for 24 h. Following incubation, the mixture was centrifuged again, and the supernatant was filtered through a 0.45 µm Millipore syringe filter. The resulting filtrate was tested for bacteriophage activity against the isolated bacteria and stored at 4 °C.

Spot assay was performed by inoculating 5 mL of sterile molten top agarose (agarose in distilled water) with 100 µL of log-phase target bacteria. The mixture was poured over a nutrient agar plate. After solidification, 5 µL of phage lysate was spotted onto the surface and allowed to absorb. Plates were incubated at 37 °C for 24 h and examined for plaques or lytic activity.

To perform double agar layer assay, phage lysate was serially diluted with SM buffer (5.8 g NaCl, 2 g MgSO_4_·7H_2_O, 50 mL 1 M Tris pH 7.5, 5 mL 2% (*w*/*v*) gelatin solution, distilled water up to 1 L). One hundred microliters of host bacterial strain and 100 µL of phage suspension from each dilution were incubated at 37 °C for 15 min to allow phage attachment. The bacteria-phage mixture was then combined with semi-solid agarose and poured onto nutrient agar plates. Plates were allowed to solidify before incubation at 37 °C for 24 h.

### 4.4. Bacteriophage Propagation, Titration, and Concentration

Clear plaques were selected, and the center of each plaque was touched with a sterile micropipette tip. The tip was placed into an Eppendorf tube containing 50 µL of SM buffer and 50 µL of 1 mM CaCl_2_, mixed by vortexing, and the double-agar layer method was repeated. This process was performed three to five times for purification. Purified plaques were stored at 4 °C. Bacteriophage titration was determined by adding an equal volume (100 µL) of serially diluted phage lysate and target bacterial culture to semi-solid top agar, mixing, and pouring over a nutrient agar base plate. Plates were cooled and incubated at 37 °C. After 24 h, plaques were counted to determine the virus titer and calculate the concentration of plaque-forming units (PFU) using the following equation:

$$\frac{pfu}{ml}=\frac{number\;of\;plaques}{volume\;of\;lysate\;infected\;with\times dilution\;factor}$$

Phage concentration was achieved through polyethylene glycol (PEG) precipitation followed by ultrafiltration. Twenty milliliters of filtered phage was treated with 10% (*w*/*v*) PEG 6000 and incubated overnight at 4 °C. The sample was then centrifuged at 5000 rpm for 25 min, the supernatant was decanted, and the pellet was resuspended in 5 mL of SM buffer containing 1 mM CaCl_2_. The concentrated phage stock was then prepared for transmission electron microscopy (TEM).

### 4.5. Molecular Identification of Bacteriophages

Genomic DNA was extracted from isolated phages (F1, B2, H3, and S4) using the FavorPrep™ Viral DNA/RNA Kit (Favorgen Biotech Corp., Ping Tung, Taiwan) according to the manufacturer’s instructions. PCR was employed to detect and identify bacteriophages in four environmental samples (01, 02, 03, and 04), targeting specific genetic markers for Myoviridae, Siphoviridae, and Podoviridae families. Four distinct primer sets were used, designed based on previously published sequences for phage classification. The primer sets are detailed in Table 2. PCR amplification was performed using GoTaq Master Mix (Promega Corporation, Madison, WI, USA). Each 25 μL reaction contained 12.5 μL of 2× GoTaq Master Mix, 1 μL of forward primer (10 μM), 1 μL of reverse primer (10 μM), 2 μL of template DNA (approximately 50 ng), and 8.5 μL of nuclease-free water. Reactions were set up in 0.2 mL thin-walled PCR tubes under sterile conditions. Negative controls (without template DNA) and positive controls (with known phage DNA) were included. Thermal cycling conditions were: initial denaturation at 95 °C for 2 min; 35 cycles of denaturation at 95 °C for 30 s, annealing at 56 °C for 30 s, and extension at 72 °C for 45 s; a final extension at 72 °C for 10 min; and a hold at 4 °C. The annealing temperature of 56 °C was optimized for all primer sets. After PCR, 5 μL of each product was mixed with 1 μL of 6× loading dye for gel electrophoresis. Remaining products were stored at −20 °C.

### 4.6. Encapsulation of Phages

All four phage stocks were diluted to 108 PFU/mL in SM buffer prior to encapsulation. To produce the alginate capsules, small-volume (10 mL) batches were formulated by extrusion of sodium alginate (Sigma-Aldrich/Merck, Darmstadt, HE, Germany) as described previously [18], with some modifications. The sodium alginate stock solution was dissolved in distilled water to a concentration of 5% (*w*/*v*) and sterilized at 121 °C for 15 min. A 5% (*w*/*v*) alginate concentration was selected after screening 2–5% formulations, as it offered the best gastric protection while still allowing sufficient phage release in simulated intestinal fluid. For capsule formation, 1 mL of each phage stock (10^8^ PFU) was added to 9 mL of the sodium alginate solution and stirred at room temperature for up to 2 min until visibly homogenized. For the extrusion method, a 22 G needle was used to eject the phage-alginate solution, dropwise, into a beaker containing 200 mL crosslinking solution (sterilized solution of CaCl_2_ 2% *w*/*v* dissolved in water) at approximately 200 rpm using a magnetic stirrer (Thermo Fisher Scientific, Waltham, MA, United States). After standing for 30 min in the crosslinking solution, the capsules were collected by decanting into a 50 mL falcon tube (Greiner Bio-One GmbH, Frickenhausen, Germany) and washed three times with 10 mL of sterile distilled water. Excess water on the surface of the capsules was capillary absorbed using a Kimwipe (Kimberly-Clark Professional, Roswell, GA, USA).

### 4.7. Encapsulation Efficiency

The phage encapsulation efficiency (EE) was determined to evaluate the titer of phage effectively encapsulated within the capsules compared to the total amount of phage used in the formulation. The EE was calculated using the following equation: EE (%) = (Pr/Pt) × 100, where Pt (PFU/mL) represents the phage titer added to the encapsulation formulation mixture and Pr (PFU/mL) represents the phage titer released from the capsules. Pr was determined using the double-agar layer method after dissolving capsules (1 g) for 20 min in 9 mL of dissolution solution (50 mM sodium citrate, 200 mM sodium hydrogen carbonate, and 50 mM Tris-HCl pH 7.5). The titer of free phage in the used crosslinking solution after encapsulation was determined using double-agar overlays.

### 4.8. Stability of Free and Encapsulated Phages

To comprehensively assess the stability of bacteriophages As, Gc, Ec, and Pa, both in their free (non-formulated) and encapsulated forms, experiments were meticulously designed to simulate a range of environmental stressors relevant to their potential applications. The methodology encompassed evaluations under varying pH conditions, temperature fluctuations, prolonged storage, and simulated gastrointestinal environments.

#### 4.8.1. pH Conditions (Simulated Gastric and Gastrointestinal Environments)

For the assessment of acidic pH conditions, mimicking the gastric environment, free phages were prepared by adding 100 µL of phage suspension (at an initial concentration of 1 × 10^6^ PFU/mL) to 900 µL of simulated gastric fluid, adjusted to specific pH levels: pH 7.5 (as control), pH 4, pH 3, pH 2.5, and pH 2. These mixtures were incubated for durations ranging from 10 min to 1 h at controlled temperature. Concurrently, freshly prepared wet capsules (1 g) containing the respective phages were subjected to the same pH values for the same incubation periods. This approach allowed for a direct comparison of phage survival in the presence and absence of encapsulation under severe acidic stress.

Furthermore, simulated gastrointestinal or intestinal conditions were employed to mimic the passage through the digestive tract. Free phage suspensions (100 µL at 1 × 10^6^ PFU/mL) underwent a sequential exposure protocol: initial incubation in simulated gastric fluid (at pH 2.0 or pH 2.5 for 1 h), followed by transfer to simulated intestinal fluids (at pH 6.0, pH 7.0, or pH 8.0, supplemented with bile salts or enzymes) for an additional 4–5 h. Encapsulated phages (1 g wet capsules) were subjected to the identical sequential exposure regimen. This sequential simulation provided critical insights into the ability of capsules to protect phages during transit through the stomach and facilitate their release in the intestinal environment.

#### 4.8.2. Temperature Variations

Temperature variations were investigated by exposing both free and encapsulated phages to a diverse set of thermal conditions. This included refrigeration (4–5 °C), ambient room temperature (21 °C), elevated heat (27 °C, 37 °C (as a control), 50 °C, 70 °C, and 80 °C) and freezing (−80 °C). For heat stability tests, phages and capsules underwent short exposures (e.g., 10, 30, 60 min) followed by a cooling period. For long-term storage stability, samples were maintained at specified temperatures (e.g., 4 °C, 21 °C) for extended periods, including 1 day, 1 week, and 1 month. This comprehensive thermal analysis aimed to elucidate the protective capacity of encapsulation against various temperature-induced stresses.

#### 4.8.3. Storage Duration and Conditions

The long-term viability of free and encapsulated phages was evaluated over 2 weeks, 1 month, and 3 months under three defined storage conditions at 4 °C and 21 °C. Free phage suspensions (100 µL at 1 × 10^6^ PFU/mL in SM buffer) and 1 g portions of freshly prepared wet capsules were tested. Dry conditions were prepared by gently blotting the wet capsules with sterile filter paper to remove excess surface water, then placing them in sealed sterile Petri dishes containing silica gel desiccant packets to maintain a low-humidity (desiccated) environment; relative humidity was not measured but remained very low due to the desiccant. Wet conditions consisted of freshly prepared (unblotted) wet capsules fully immersed in sterile SM buffer containing 1 mM CaCl_2_ (to preserve cross-linking) in sealed sterile tubes. Humid conditions involved gently blotted but still hydrated capsules placed in sealed sterile Petri dishes without desiccant, stored at 21 °C under ambient laboratory relative humidity (approximately 50–70% RH at the time of the experiment); no active humidity control chamber was used. For encapsulated phages, physical integrity of the capsules was periodically examined by optical microscopy to monitor visible changes (e.g., swelling, disintegration, or deformation) and to correlate structural stability with phage viability.

#### 4.8.4. Phage Titre Determination

Following exposure to each respective environmental stress condition, the phage titre was determined. For free phages, the titre was directly measured using double-agar overlays. For encapsulated phages, the capsules were first separated from the stress solution, thoroughly washed with sterile water, and then allowed to disintegrate in 10 mL SM buffer for 1 h at 37 °C with continuous shaking at 150 rpm. The titre of the released phages was subsequently quantified using the double-agar overlay plaque assay. This rigorous and detailed experimental framework allows for a comprehensive evaluation of phage stability and the protective effects afforded by encapsulation under a wide array of relevant conditions, thereby informing their potential for diverse practical applications.

### 4.9. Morphology of the Phage Capsules

The size and surface morphology of the capsules were examined using a field emission scanning electron microscope (SEM; JEOL JSM-7610F, JEOL Ltd., Tokyo, Japan) and macroscopic digital photography with a Canon EOS 70D digital camera (Ōita, Japan) equipped with a 100 mm macro lens. Wet and dry capsules were mounted on metal stubs with double-sided conductive tape and sputter-coated with a thin gold/palladium (Au/Pd) layer under vacuum to enhance conductivity and prevent charging artifacts during imaging.

### 4.10. Statistical Analysis

All statistical analyses were conducted using GraphPad Prism software (version 9.0 or higher; GraphPad Software, San Diego, CA, USA). Phage titers were log_10_-transformed and reported as mean ± standard deviation (SD) from at least three independent replicates (*n* ≥ 3). Comparisons between free and encapsulated phages were performed using unpaired two-tailed Student’s t-tests. Multiple-group comparisons (e.g., across pH, temperature, or time) employed one-way or two-way ANOVA followed by Tukey’s or Sidak’s post hoc tests, as appropriate. Normality was checked using the Shapiro–Wilk test; non-parametric tests (Mann–Whitney U or Kruskal–Wallis) were used when necessary. Statistical significance was set at *p* < 0.05.

## 5. Conclusions

In conclusion, this study highlights the immense potential of wastewater-derived bacteriophages as effective and sustainable biocontrol agents against diverse bacterial pathogens, including *A. hydrophila*, *E. coli*, *P. aeruginosa*, and *G. creatinolyticus*. Sodium alginate encapsulation successfully addressed primary technical hurdles in phage therapy: extreme vulnerability to gastric acidity and cold-chain storage requirements. By providing a protective microenvironment, the alginate matrix ensured therapeutic phage titer survival through simulated gastrointestinal transit and maintained high viability at ambient temperatures for extended periods. These findings significantly contribute to the growing evidence supporting standardized, formulated phage products for oral administration in veterinary and human medicine. The transition from free phage suspensions to stable, encapsulated delivery systems represents a critical step toward clinical translation. Ultimately, integrating robust phage isolation protocols with advanced encapsulation technologies offers a powerful strategy for combating the global crisis of antimicrobial resistance, providing a versatile and adaptable alternative to traditional antibiotics.

## Figures and Tables

**Table 1:** Viability of unencapsulated and encapsulated bacteriophages As, Gc, Ec, and Pa under different pH, temperature, and storage conditions.

Condition	Phage	Unencaps. (PFU/mL)	SD	Encap. (PFU/mL)	SD	* *p*-Value
pH 2.0 (10 min)	As	<1.0 × 10^1^	0	1.16 × 10^3^	±147	<0.001
	Gc	0	0	2.16 × 10^3^	±154	<0.001
	Ec	<1.0 × 10^1^	0	2.70 × 10^3^	±395	<0.001
	Pa	0	0	1.90 × 10^3^	±157	<0.001
pH 2.5 (10 min)	As	0	0	4.00 × 10^3^	±310	<0.001
	Gc	1.0 × 10^1^	±2	2.00 × 10^3^	±122	<0.001
	Ec	1.0 × 10^1^	±1	1.00 × 10^4^	±1.5 × 10^3^	<0.001
	Pa	0	0	3.00 × 10^3^	±417	<0.001
pH 2.0 (60 min)	All	≤1.0 × 10^1^	0	1.58 × 10^2^	±6	<0.001
pH 3.0 (10 min)	All	3.16 × 10^5^	±28.6 × 10^3^	1.00 × 10^6^	±118 × 10^3^	<0.01
pH 7.5 (control)	All	1.00 × 10^6^	±83 × 10^3^	1.00 × 10^6^	±148 × 10^3^	>0.05
pH 10	As	1.31 × 10^2^	±17	1.40 × 10^3^	±93	<0.001
	Gc	2.00 × 10^2^	±16	2.50 × 10^3^	±335	<0.001
	Ec	2.33 × 10^2^	±14	3.10 × 10^3^	±802	<0.001
	Pa	1.33 × 10^2^	±22	1.40 × 10^2^	±10	>0.05
pH 13	As	1.50 × 10^1^	±2	1.90 × 10^2^	±20	<0.001
	Gc	3.15 × 10^1^	±4	4.27 × 10^2^	±34	<0.001
	Ec	<1.0 × 10^1^	0	3.93 × 10^1^	±1	<0.01
	Pa	1.00 × 10^1^	±1	1.28 × 10^1^	±1	>0.05
Simulated GI (SGF + SIF)	All	≤1.0 × 10^1^	0	3.16 × 10^2^	±36	<0.001
50 °C (30 min)	As	2.22 × 10^2^	±32	2.22 × 10^3^	±326	<0.001
	Gc	7.33 × 10^1^	±12	7.33 × 10^2^	±162	<0.001
	Ec	1.22 × 10^2^	±4	2.40 × 10^3^	±162	<0.001
	Pa	2.43 × 10^1^	±4	3.00 × 10^2^	±30	<0.001
70 °C (10 min)	As	1.0 × 10^1^	±1	3.16 × 10^1^	±4	<0.001
	Gc	1.0 × 10^1^	±1	3.16 × 10^1^	±4	<0.001
	Ec	5.53 × 10^1^	±7	5.53 × 10^2^	±38	<0.001
	Pa	1.0 × 10^1^	±2	3.16 × 10^1^	±2	<0.001
80 °C (10 min)	All	≤3.3 × 10^1^	±6	≤3.3 × 10^2^	±39	>0.05
4 °C wet storage (3 months)	All	6.31 × 10^4^	±8.4 × 10^3^	1.58 × 10^5^	±14.8 × 10^3^	<0.05
21 °C dry (3 months)	All	1.00 × 10^6^	±42 × 10^3^	1.00 × 10^6^	±72 × 10^3^	>0.05
21 °C humid (3 months)	All	1.00 × 10^6^	±51 × 10^3^	1.00 × 10^6^	±183 × 10^3^	>0.05

* *p*-values indicate the statistical comparison between encapsulated and unencapsulated phages for each individual phage and condition (unpaired *t*-test). Significance levels: * *p* < 0.05, ns = not significant (*p* > 0.05).

**Table 2:** List of primers used in the current study.

No.	Primer	Family	Sequence	TM (°C)	Amplicon Size (bp)
1	G23-F	Myoviridae	5′-ACWGGWCTKATYTTCGCAATG-3′	57.3	568
	G23-R		5′-AYTTYTCAACWGACCADCKACC-3′	59.5	
2	G43-F		5′-GCWGGTGCWTATGTHAARGAACC-3′	60.3	476
	G43-R		5′-CCWGASARAGTAATKGCYTCWGC-3′	61.9	
3	Siph-F	Siphoviridae	5′-GCGTGATGGTTGGGATGGTA-3′	62.8	459
	Siph-R		5′-GACGCTCAATCTGACGACCA-3′	62	
4	Pod-F	Podoviridae	5′-CCGCGATTGCGAGCATTAAA-3′	59.6	774
	Pod-R		5′-CGGTCTGAATGTTCACCGGA-3′	63.1	

## Data Availability

Data is contained within the article or Supplementary Materials.

## Supplementary Materials

The following supporting information can be downloaded at: https://www.mdpi.com/article/10.3390/ph19030363/s1. Supplementary Figures provide additional images for plaque assay (Figure S1–S7) and TEM images for Phage As, Phage Pa, Phage Gc, and Phage Ec (Figures S8–S18), digital camera images of phage capsules (Figures S19–S24), SEM images of dry and wet capsules (Figure S25–S30).

## Abbreviations

The following abbreviations are used in this manuscript:

AMR	Antimicrobial Resistance
EE	Encapsulation Efficiency
GIT	Gastrointestinal Tract
JEM	JEOL Electron Microscope (model)
MOI	Multiplicity of Infection
OD	Optical Density
PFU	Plaque Forming Units
SEM	Scanning Electron Microscopy
SGF	Simulated Gastric Fluid
SIF	Simulated Intestinal Fluid
TEM	Transmission Electron Microscopy
WGS	Whole-Genome Sequencing
WWTP	Wastewater Treatment Plant
