# Supplementary figures and images for: Encapsulation of Bacteriophages in Alginate Beads: Improved Viability Under Harsh Simulated Gastric and Intestinal Conditions for Phage Therapy Applications
Source: Pharmaceuticals (Basel). 2026 Feb 25;19(3):363. doi: 10.3390/ph19030363 (PMC13029702; doi:10.3390/ph19030363)

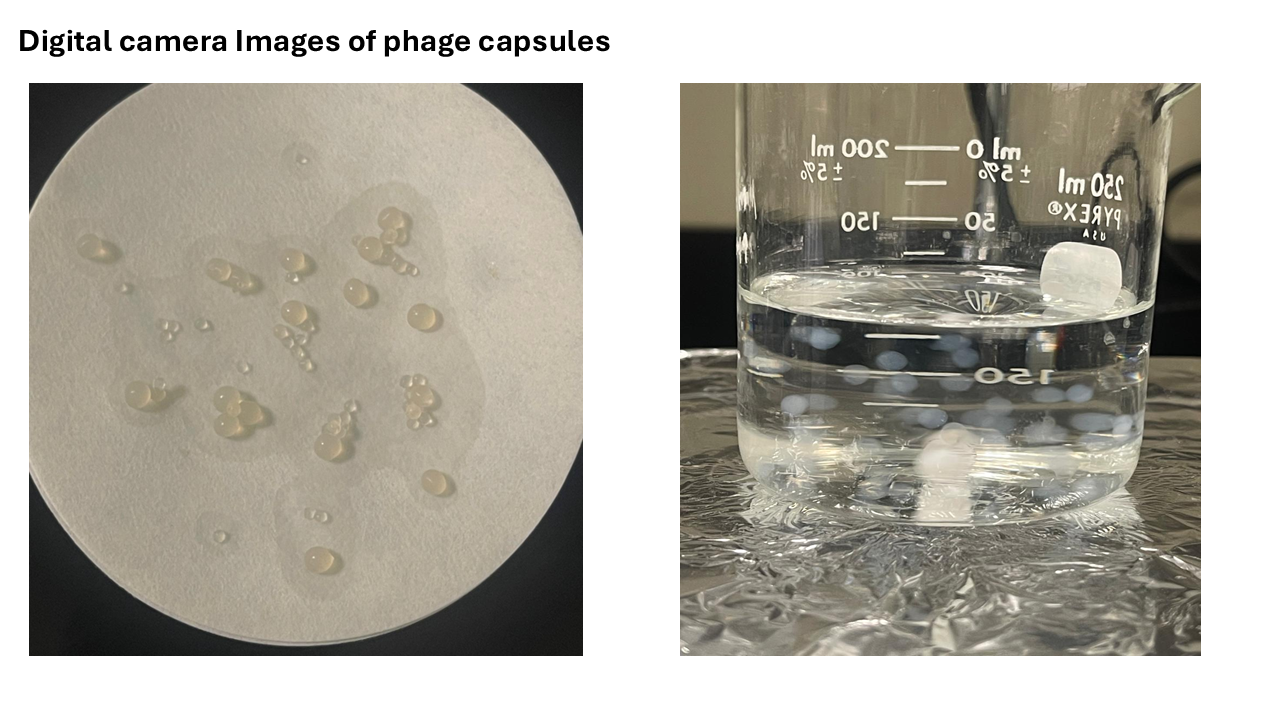

Supplement: Supplementary file 1 [file pharmaceuticals-19-00363-s001.zip › pharmaceuticals-4126957-supplementary File S1/Digital camera images of phage capsules/Figure S19. Digital camera images of phage capsules-01.TIF]

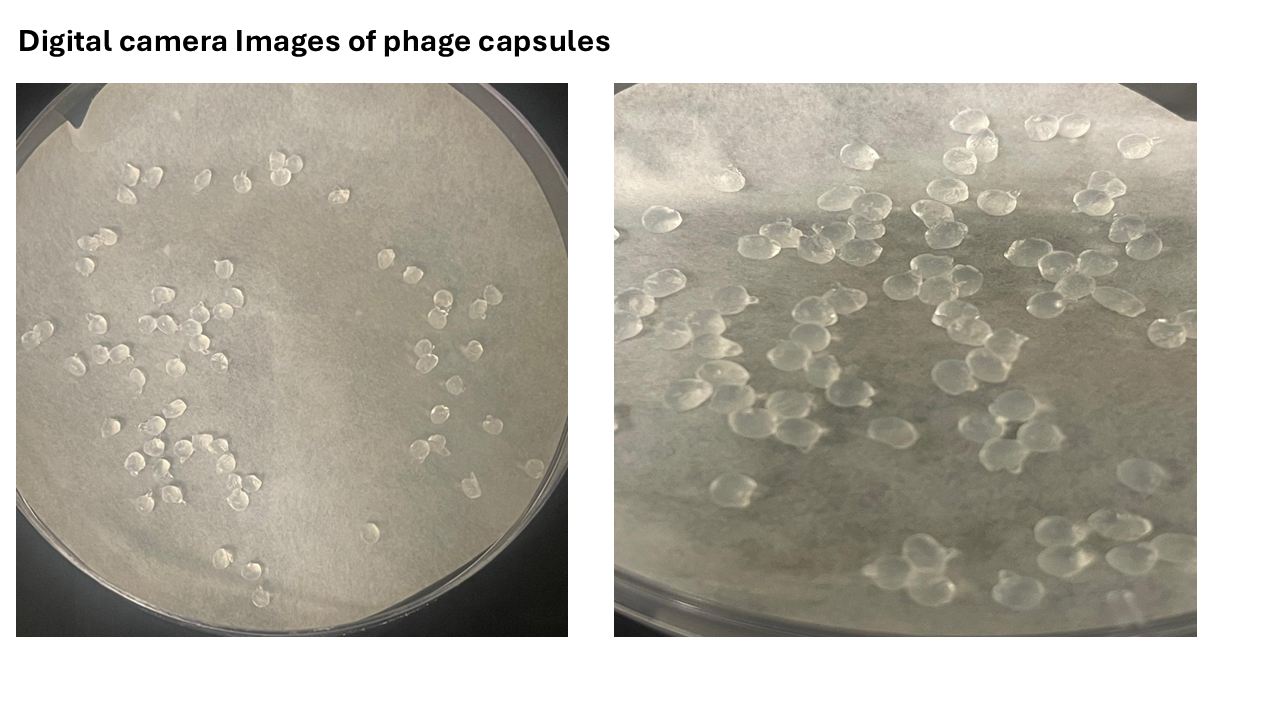

Supplement: Supplementary file 1 [file pharmaceuticals-19-00363-s001.zip › pharmaceuticals-4126957-supplementary File S1/Digital camera images of phage capsules/Figure S20. Digital camera images of phage capsules-02.TIF]

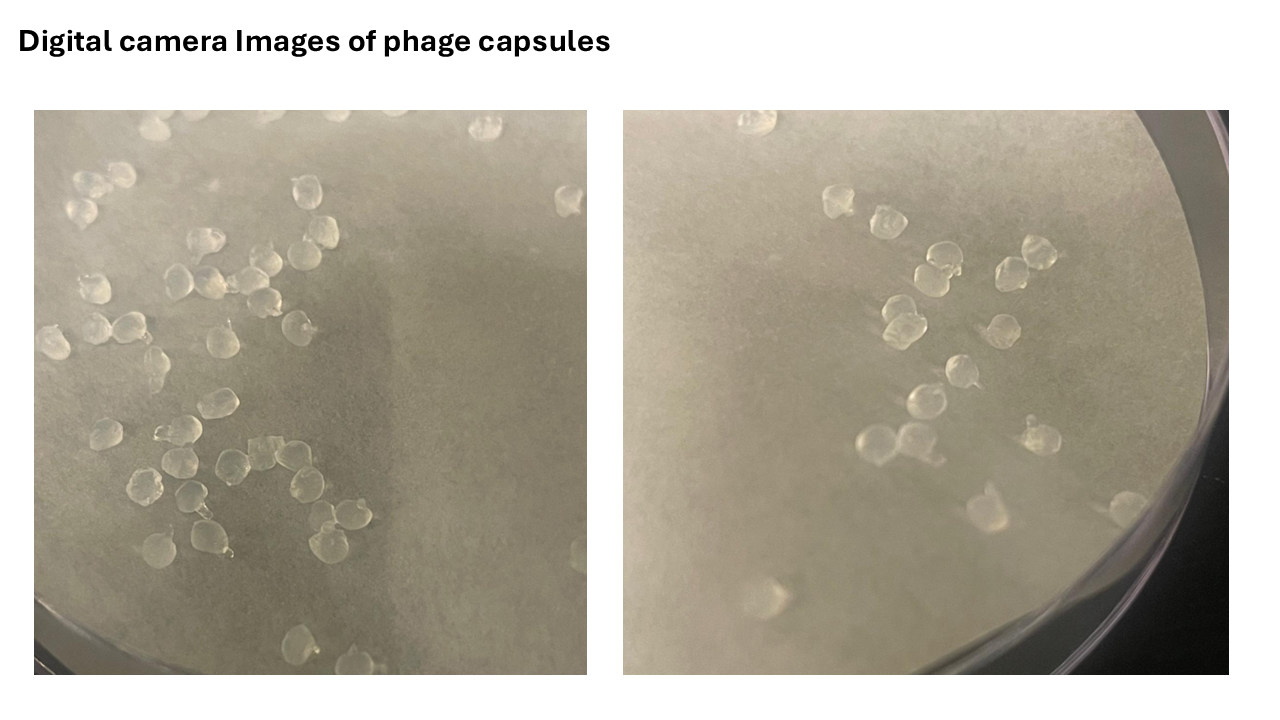

Supplement: Supplementary file 1 [file pharmaceuticals-19-00363-s001.zip › pharmaceuticals-4126957-supplementary File S1/Digital camera images of phage capsules/Figure S21. Digital camera images of phage capsules-03.TIF]

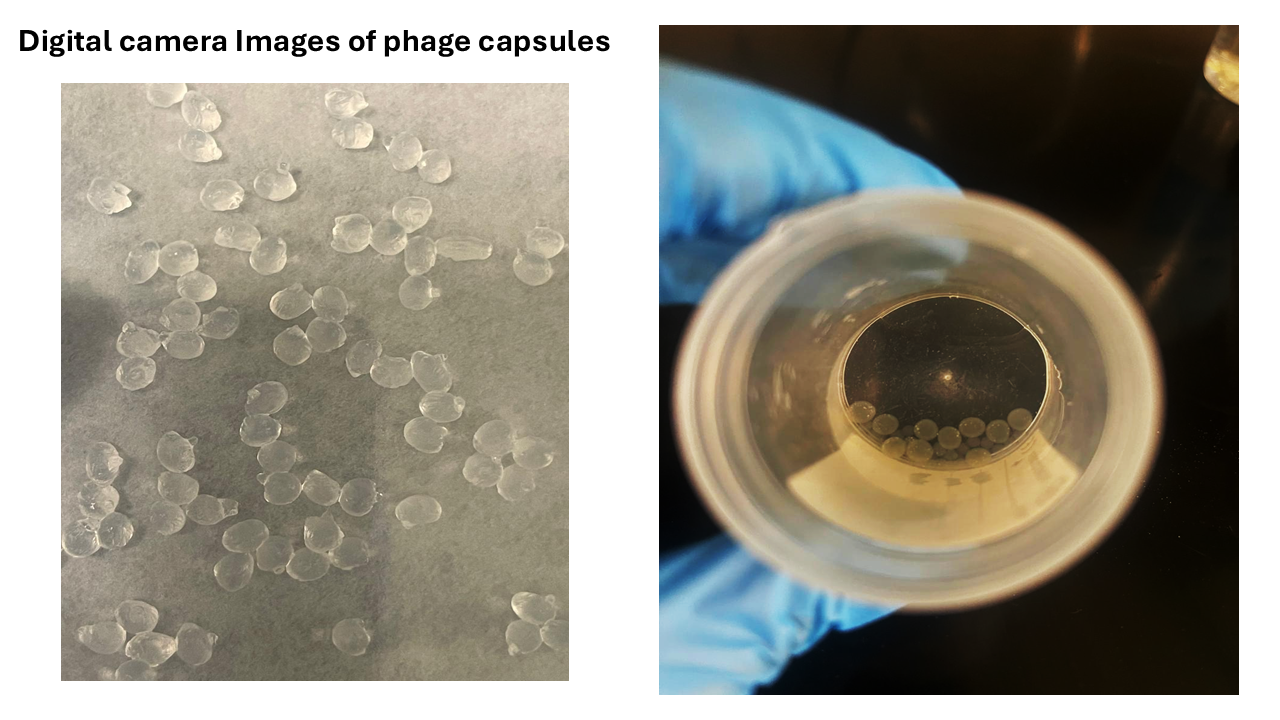

Supplement: Supplementary file 1 [file pharmaceuticals-19-00363-s001.zip › pharmaceuticals-4126957-supplementary File S1/Digital camera images of phage capsules/Figure S22. Digital camera images of phage capsules-04.TIF]

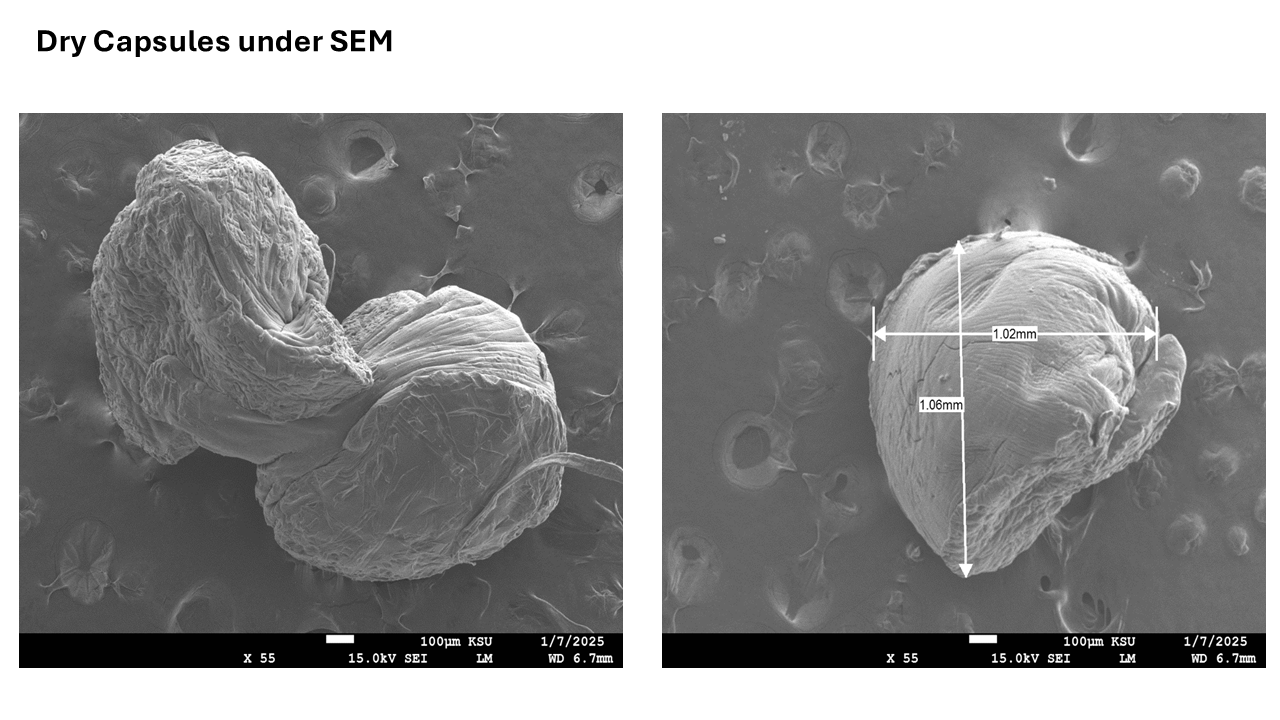

Supplement: Supplementary file 1 [file pharmaceuticals-19-00363-s001.zip › pharmaceuticals-4126957-supplementary File S1/Dry and Wet Capsules Under SEM/Figure S25. Dry Capsules-SEM images-01.TIF]

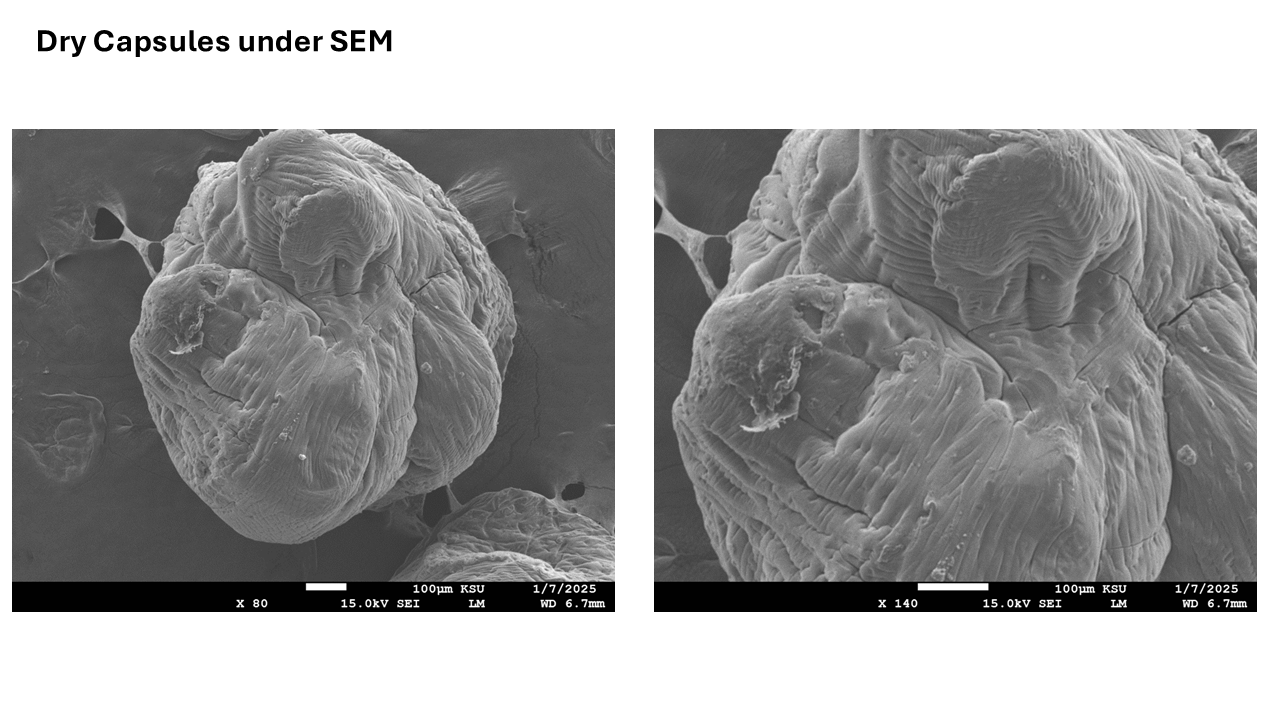

Supplement: Supplementary file 1 [file pharmaceuticals-19-00363-s001.zip › pharmaceuticals-4126957-supplementary File S1/Dry and Wet Capsules Under SEM/Figure S26. Dry Capsules-SEM images-02.TIF]

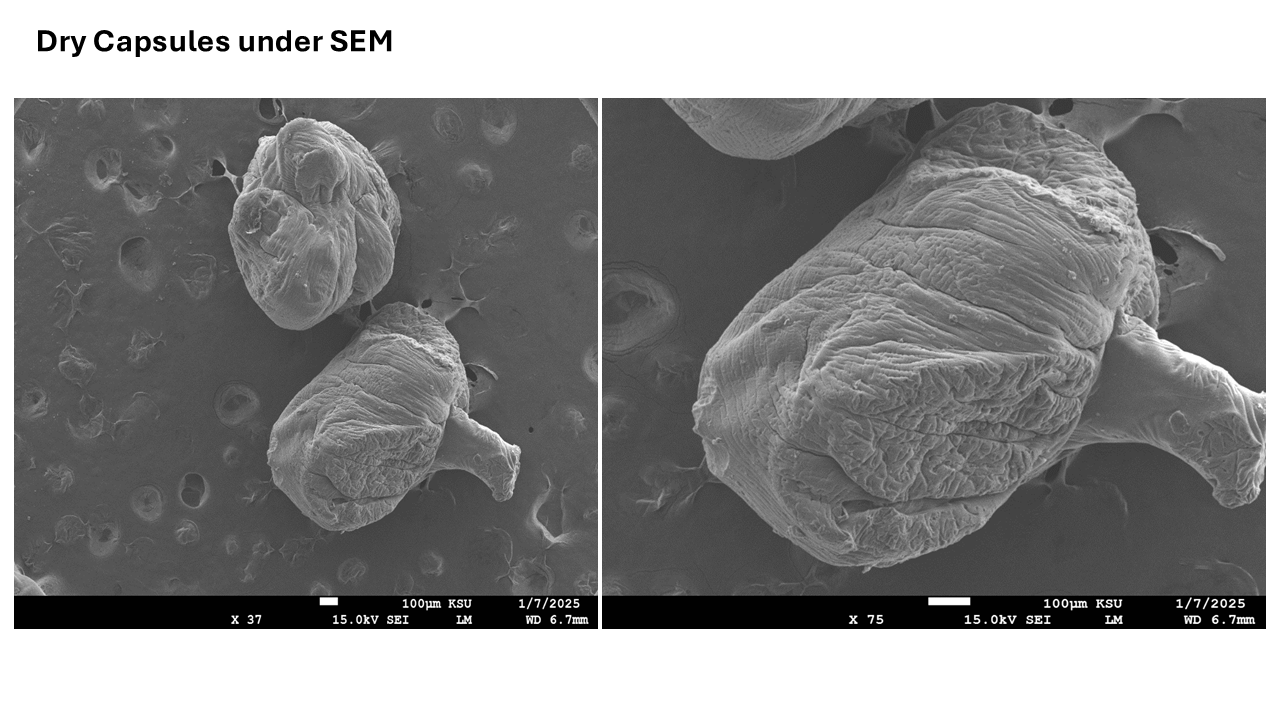

Supplement: Supplementary file 1 [file pharmaceuticals-19-00363-s001.zip › pharmaceuticals-4126957-supplementary File S1/Dry and Wet Capsules Under SEM/Figure S27. Dry Capsules-SEM images-03.TIF]

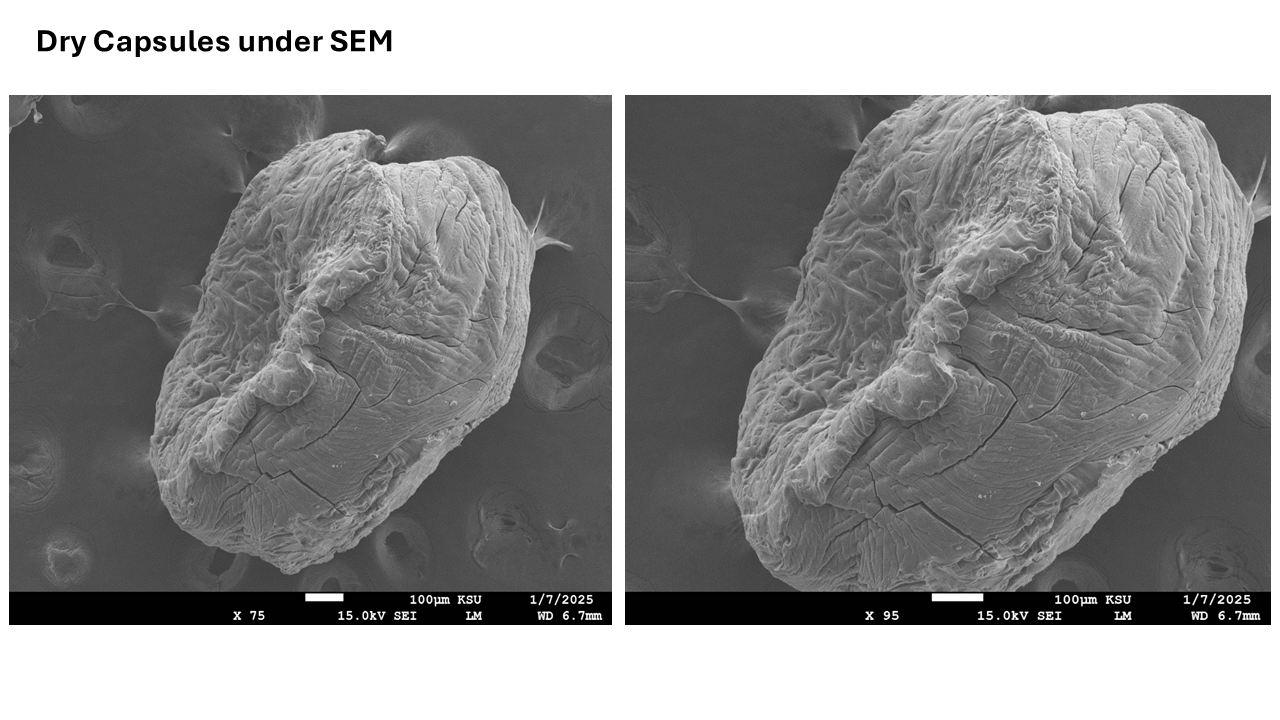

Supplement: Supplementary file 1 [file pharmaceuticals-19-00363-s001.zip › pharmaceuticals-4126957-supplementary File S1/Dry and Wet Capsules Under SEM/Figure S28. Dry Capsules-SEM images-04.TIF]

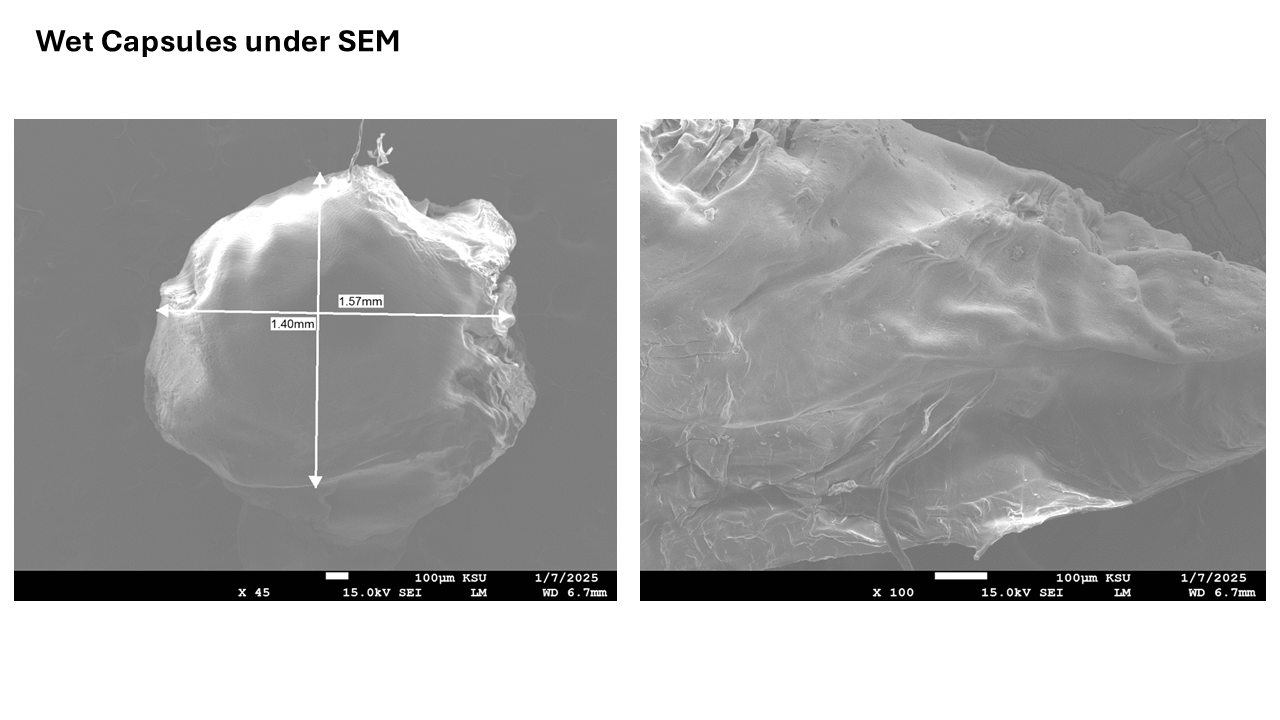

Supplement: Supplementary file 1 [file pharmaceuticals-19-00363-s001.zip › pharmaceuticals-4126957-supplementary File S1/Dry and Wet Capsules Under SEM/Figure S29. Wet Capsules-SEM images-01.TIF]

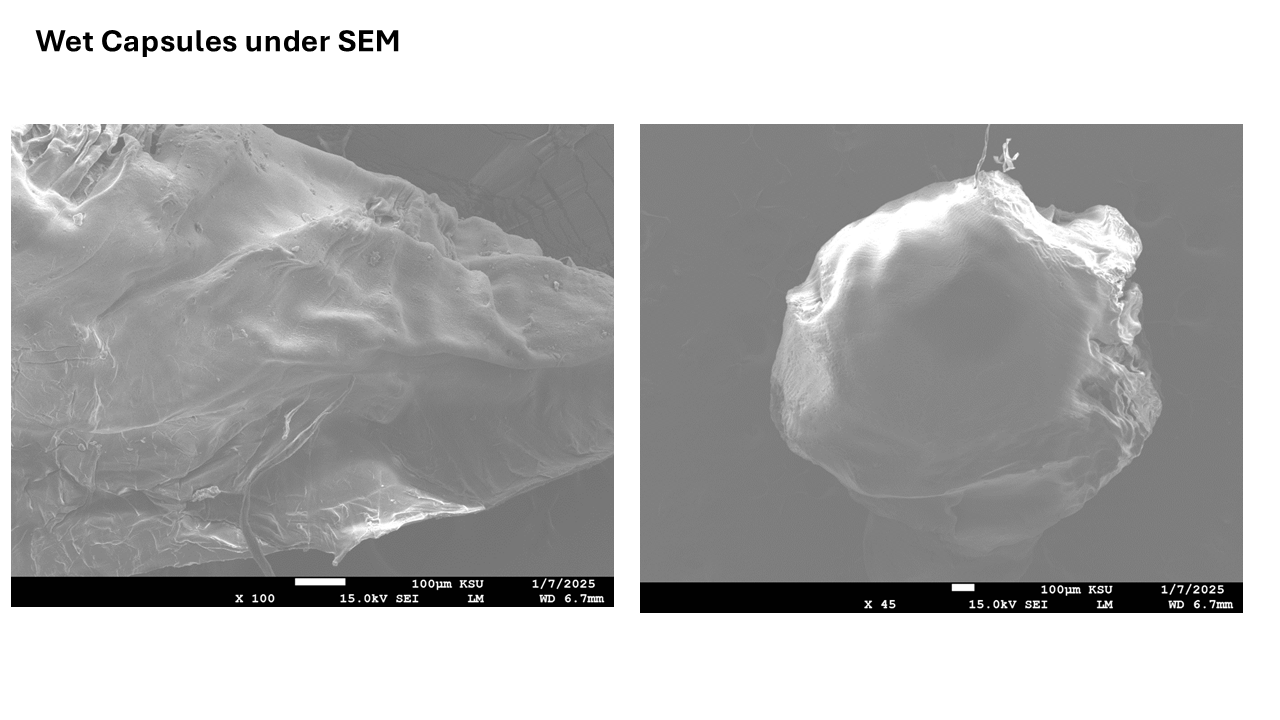

Supplement: Supplementary file 1 [file pharmaceuticals-19-00363-s001.zip › pharmaceuticals-4126957-supplementary File S1/Dry and Wet Capsules Under SEM/Figure S30. Wet Capsules-SEM images-02.TIF]

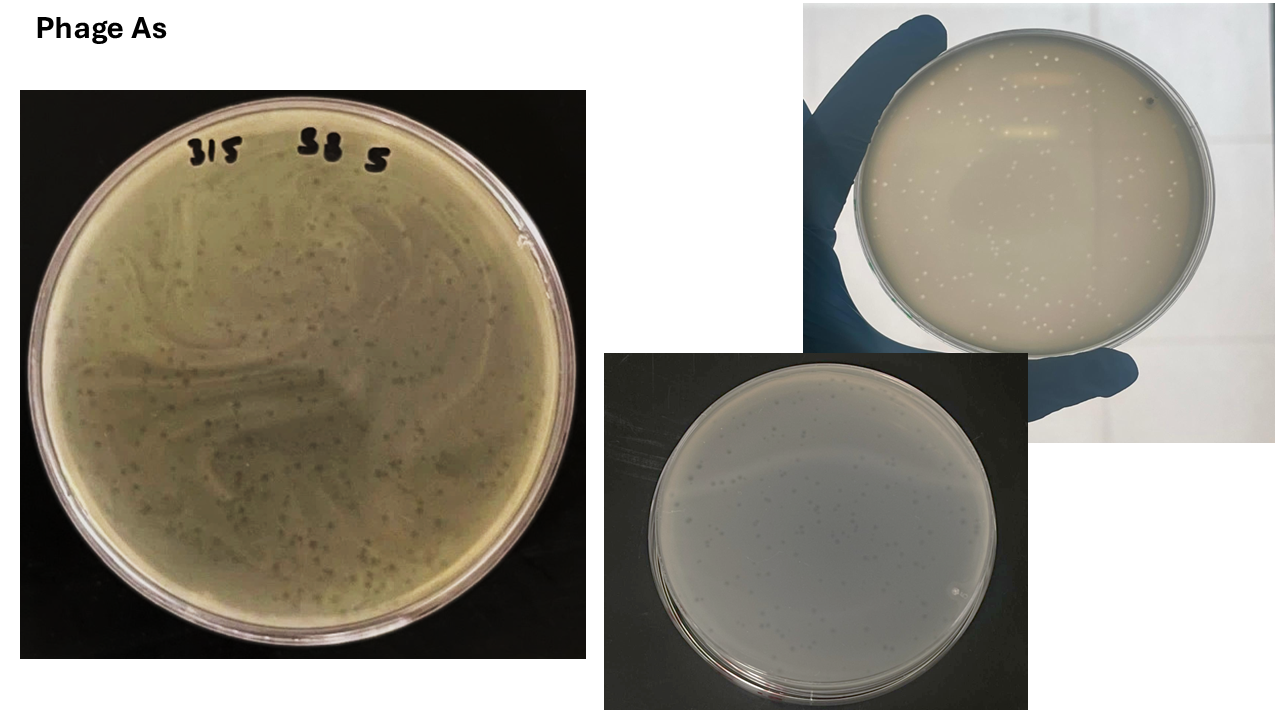

Supplement: Supplementary file 1 [file pharmaceuticals-19-00363-s001.zip › pharmaceuticals-4126957-supplementary File S1/Phage As/Figure S1. Phage As-Plaque Assay.TIF]

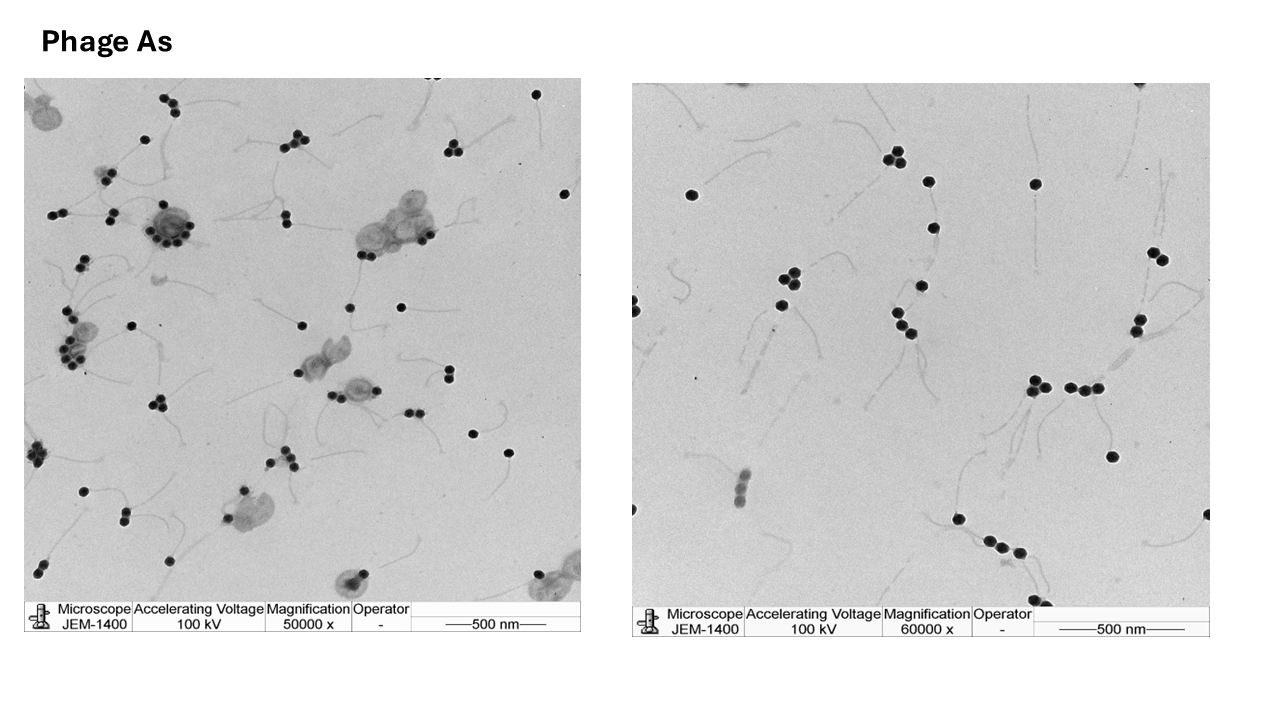

Supplement: Supplementary file 1 [file pharmaceuticals-19-00363-s001.zip › pharmaceuticals-4126957-supplementary File S1/Phage As/Figure S10. TEM-image-03-Phage As.TIF]

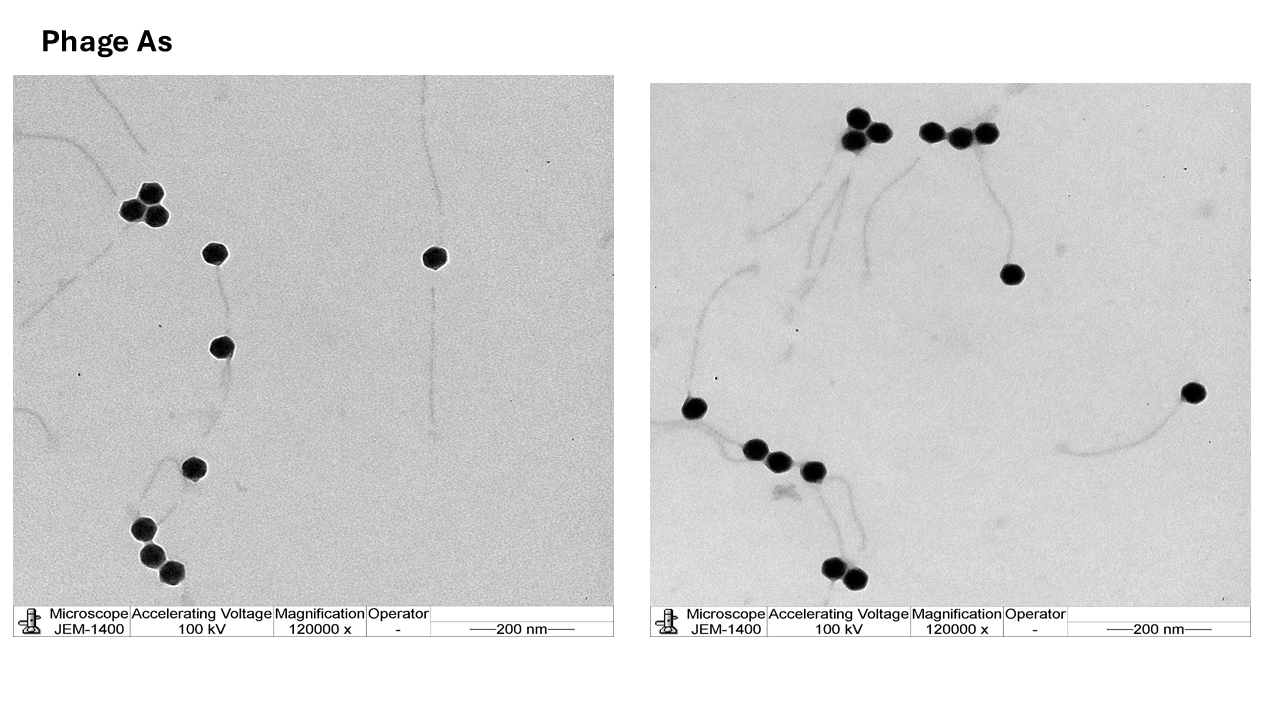

Supplement: Supplementary file 1 [file pharmaceuticals-19-00363-s001.zip › pharmaceuticals-4126957-supplementary File S1/Phage As/Figure S11. TEM-image-04.TIF]

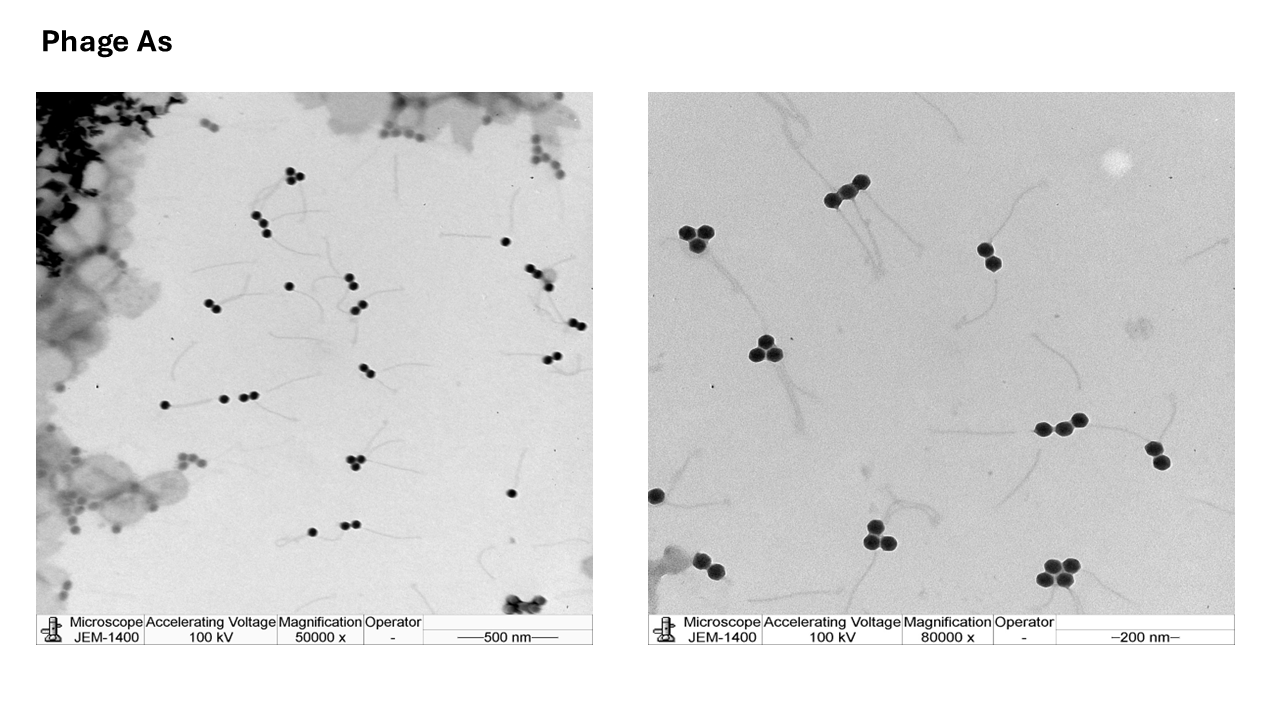

Supplement: Supplementary file 1 [file pharmaceuticals-19-00363-s001.zip › pharmaceuticals-4126957-supplementary File S1/Phage As/Figure S8. TEM-image-01-Phage As.TIF]

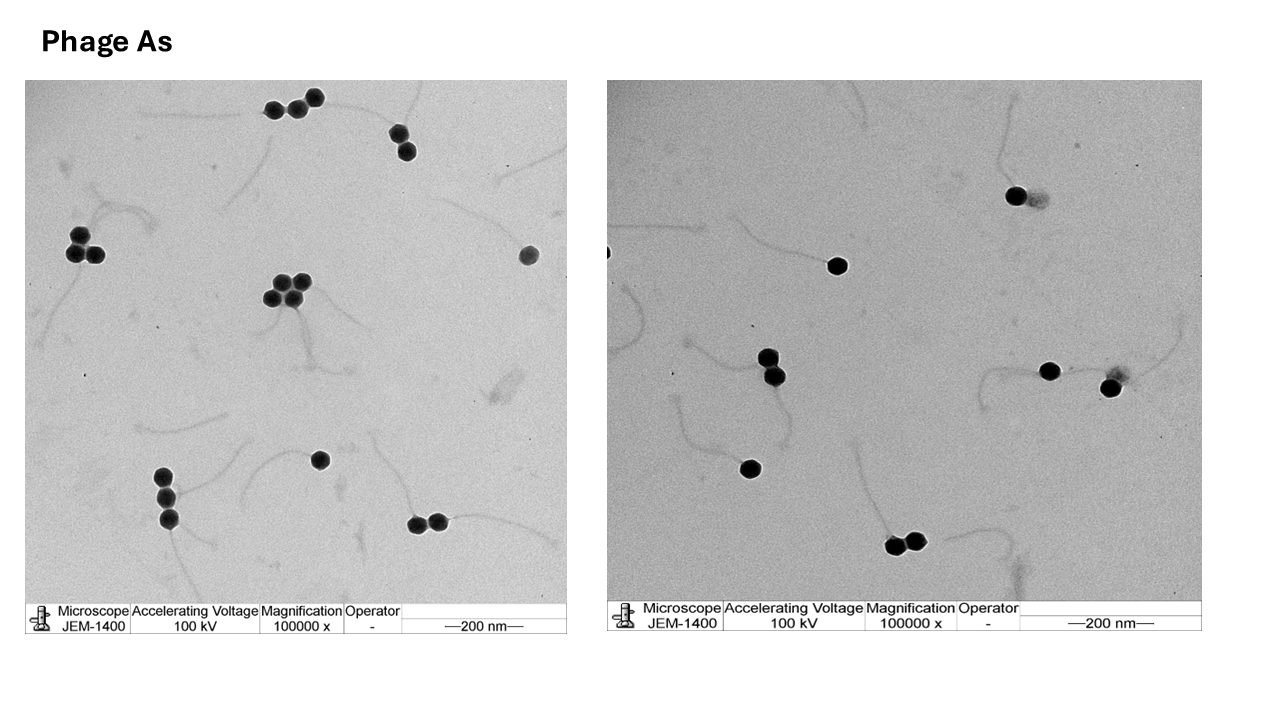

Supplement: Supplementary file 1 [file pharmaceuticals-19-00363-s001.zip › pharmaceuticals-4126957-supplementary File S1/Phage As/Figure S9. TEM-image-02-Phage As.TIF]

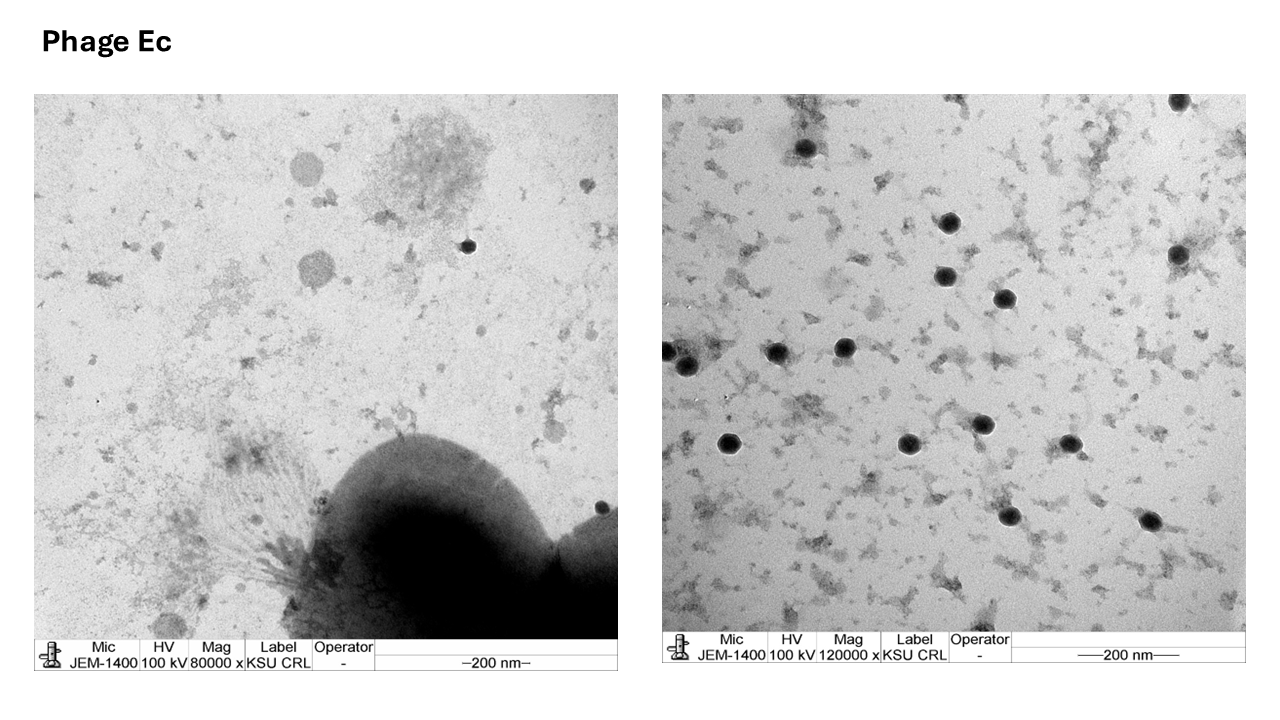

Supplement: Supplementary file 1 [file pharmaceuticals-19-00363-s001.zip › pharmaceuticals-4126957-supplementary File S1/Phage Ec/Figure S12. TEM-image-01-Phage Ec.TIF]

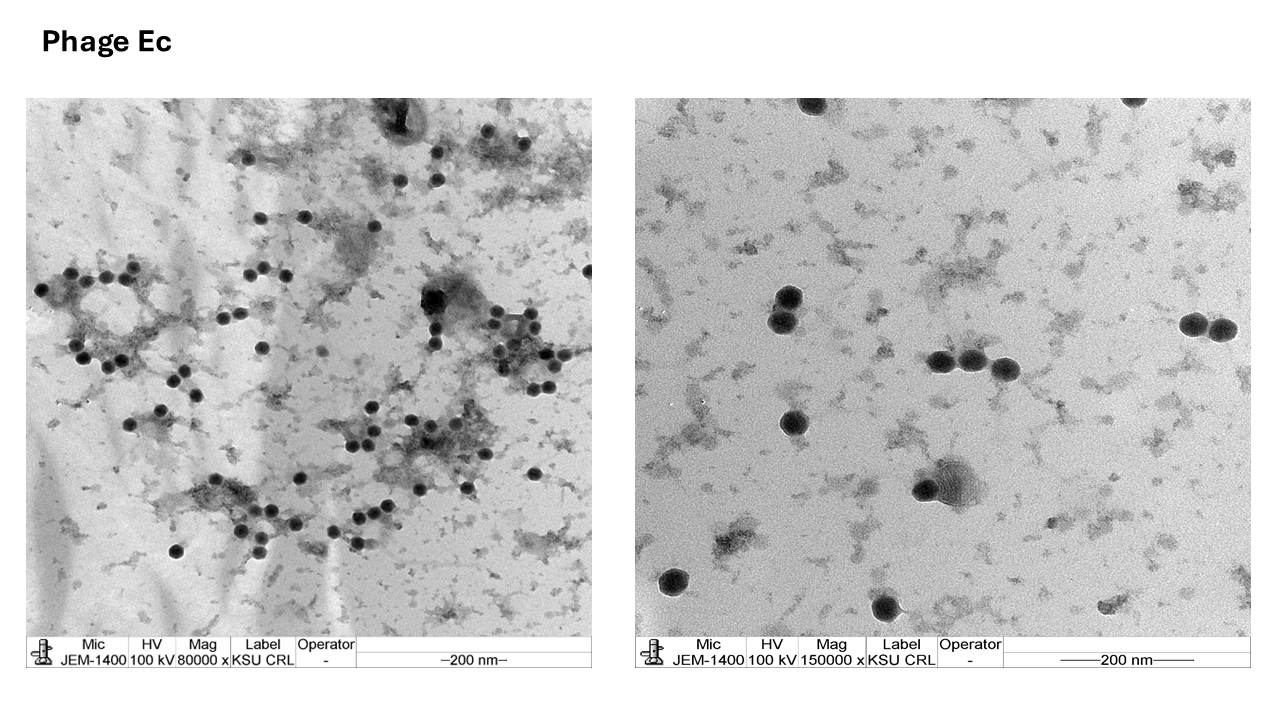

Supplement: Supplementary file 1 [file pharmaceuticals-19-00363-s001.zip › pharmaceuticals-4126957-supplementary File S1/Phage Ec/Figure S13. TEM-image-02-Phage Ec.TIF]

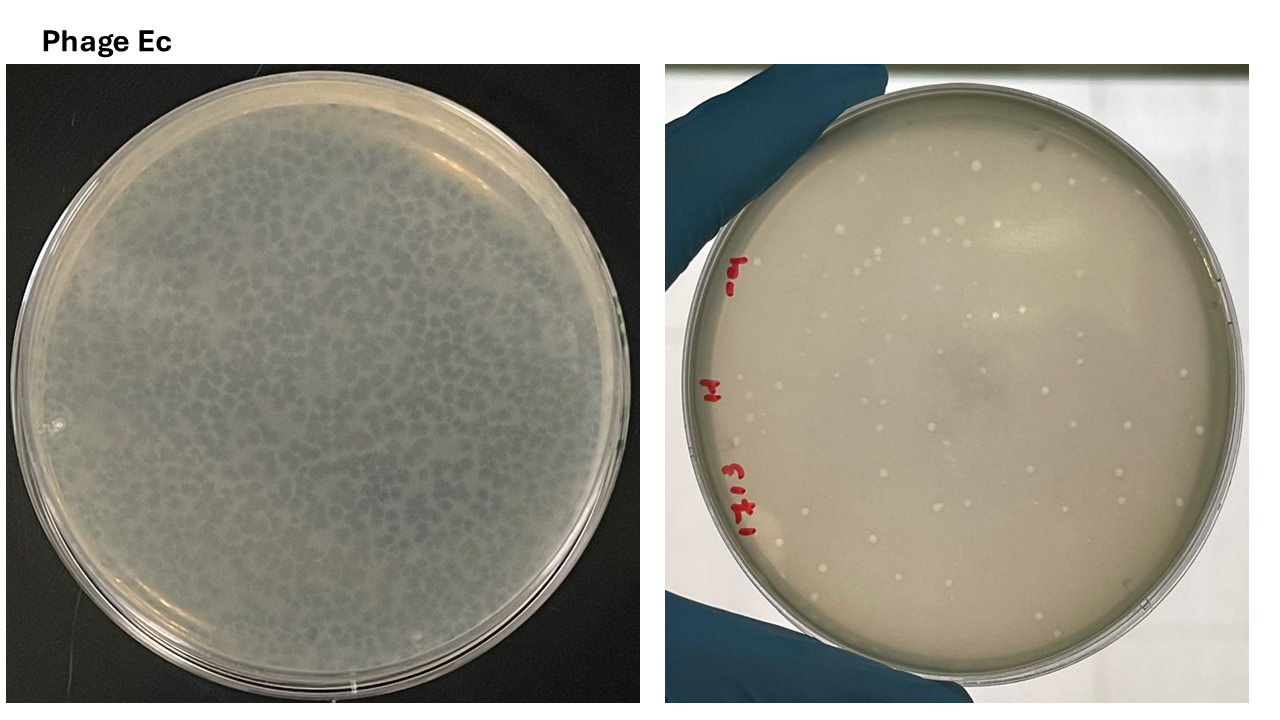

Supplement: Supplementary file 1 [file pharmaceuticals-19-00363-s001.zip › pharmaceuticals-4126957-supplementary File S1/Phage Ec/Figure S2. Phage Ec-Plaque Assay.TIF]

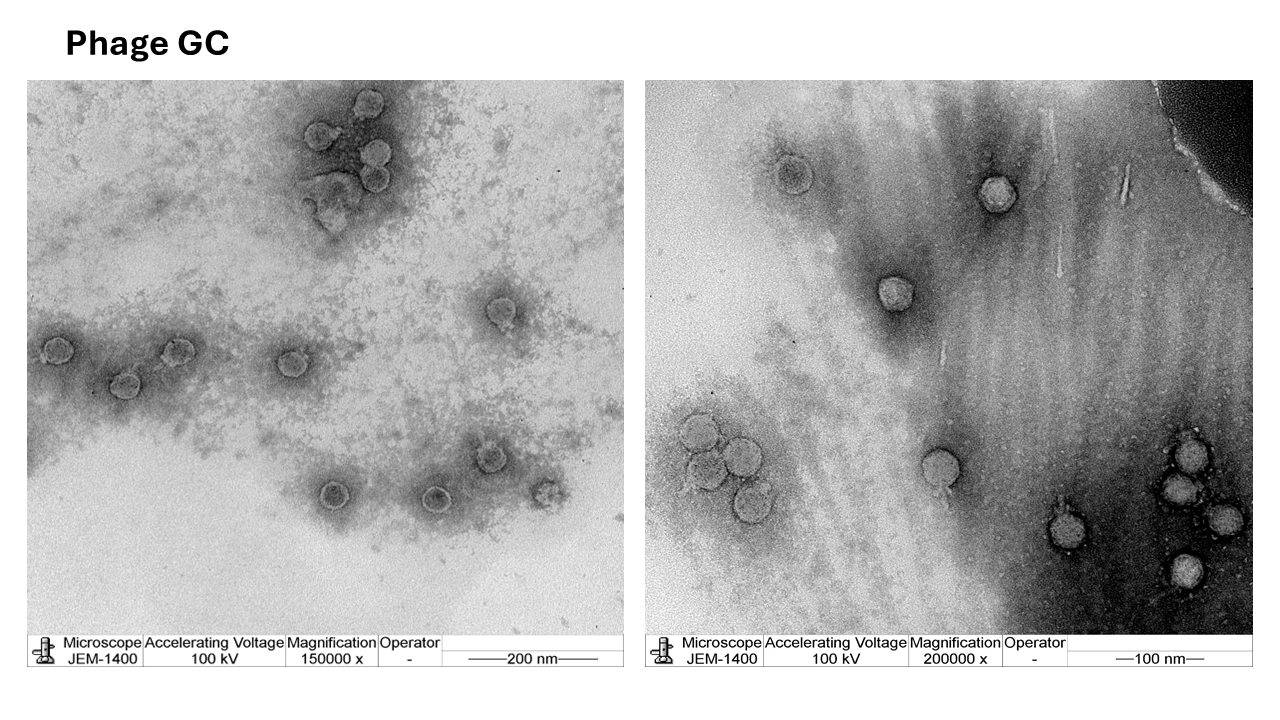

Supplement: Supplementary file 1 [file pharmaceuticals-19-00363-s001.zip › pharmaceuticals-4126957-supplementary File S1/Phage GC/Figure S14. TEM-image-01-Phage Gc.TIF]

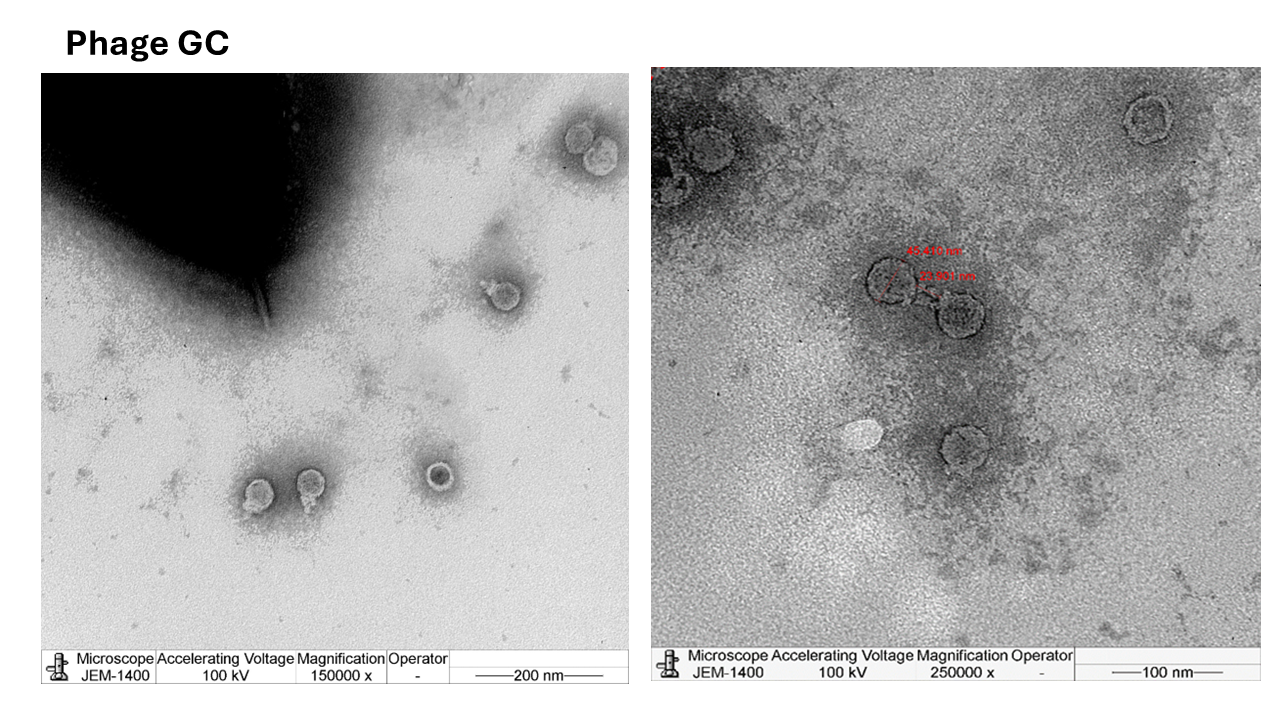

Supplement: Supplementary file 1 [file pharmaceuticals-19-00363-s001.zip › pharmaceuticals-4126957-supplementary File S1/Phage GC/Figure S15. TEM-image-02-Phage Gc.TIF]

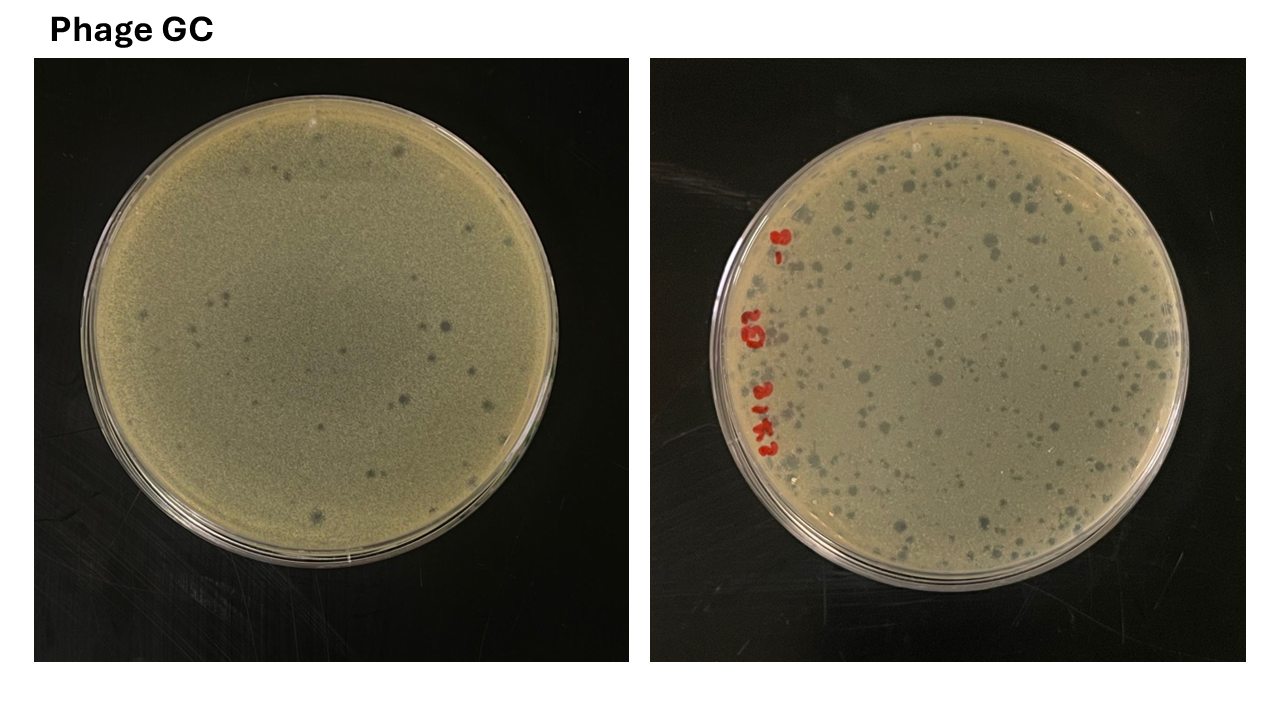

Supplement: Supplementary file 1 [file pharmaceuticals-19-00363-s001.zip › pharmaceuticals-4126957-supplementary File S1/Phage GC/Figure S3. PhageGc-Plaque Assay-01.TIF]

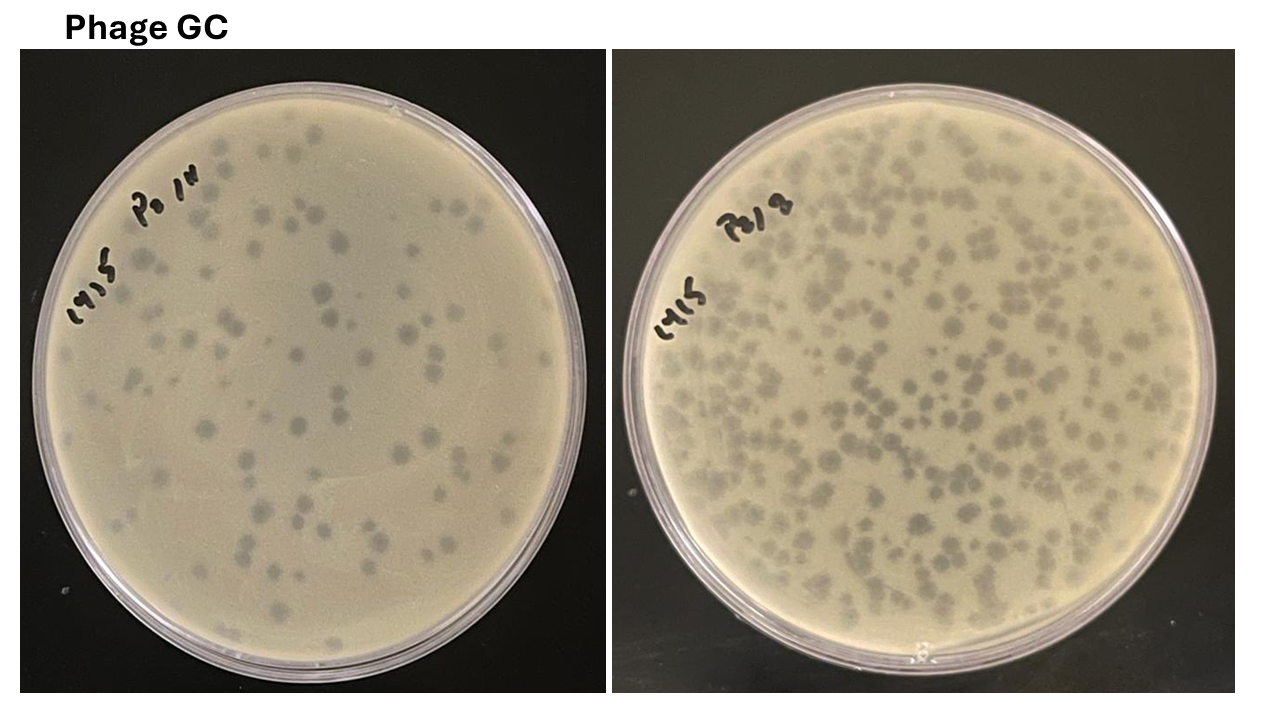

Supplement: Supplementary file 1 [file pharmaceuticals-19-00363-s001.zip › pharmaceuticals-4126957-supplementary File S1/Phage GC/Figure S4. PhageGc-Plaque Assay-02.TIF]

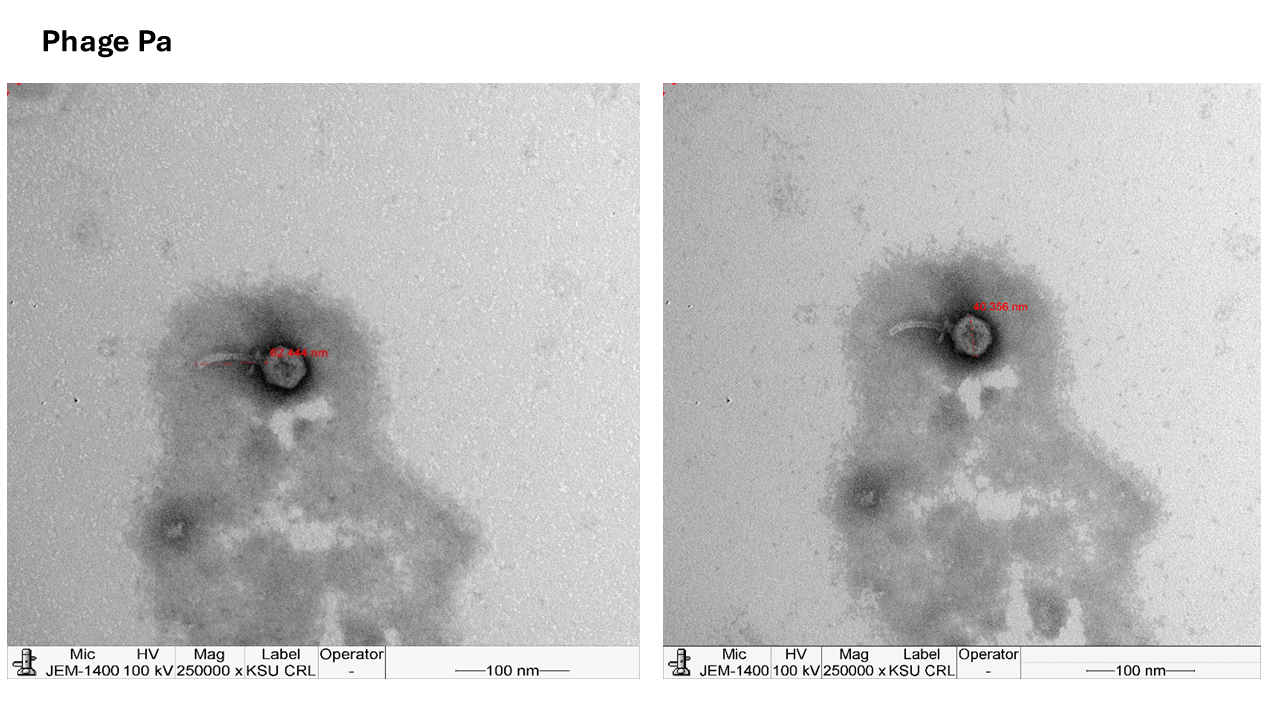

Supplement: Supplementary file 1 [file pharmaceuticals-19-00363-s001.zip › pharmaceuticals-4126957-supplementary File S1/Phage Pa/Figure S16. TEM-image-01-Phage Pa.TIF]

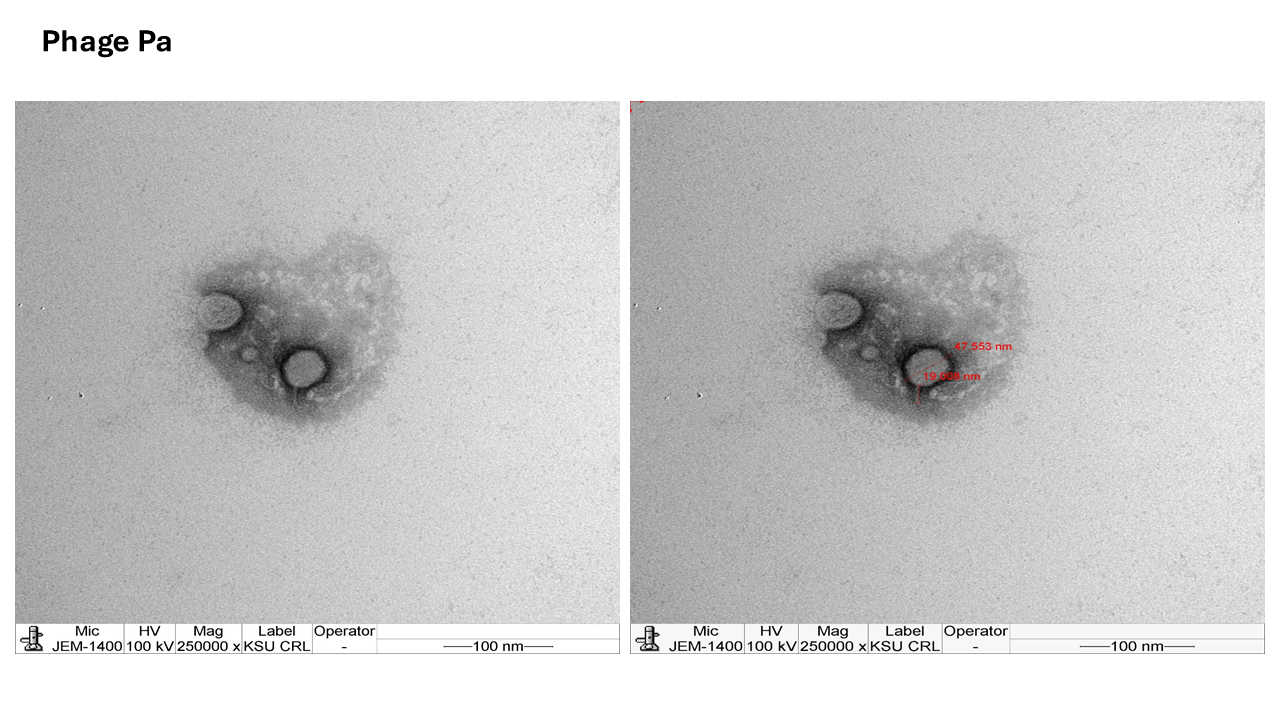

Supplement: Supplementary file 1 [file pharmaceuticals-19-00363-s001.zip › pharmaceuticals-4126957-supplementary File S1/Phage Pa/Figure S17. TEM-image-02-Phage Pa.TIF]

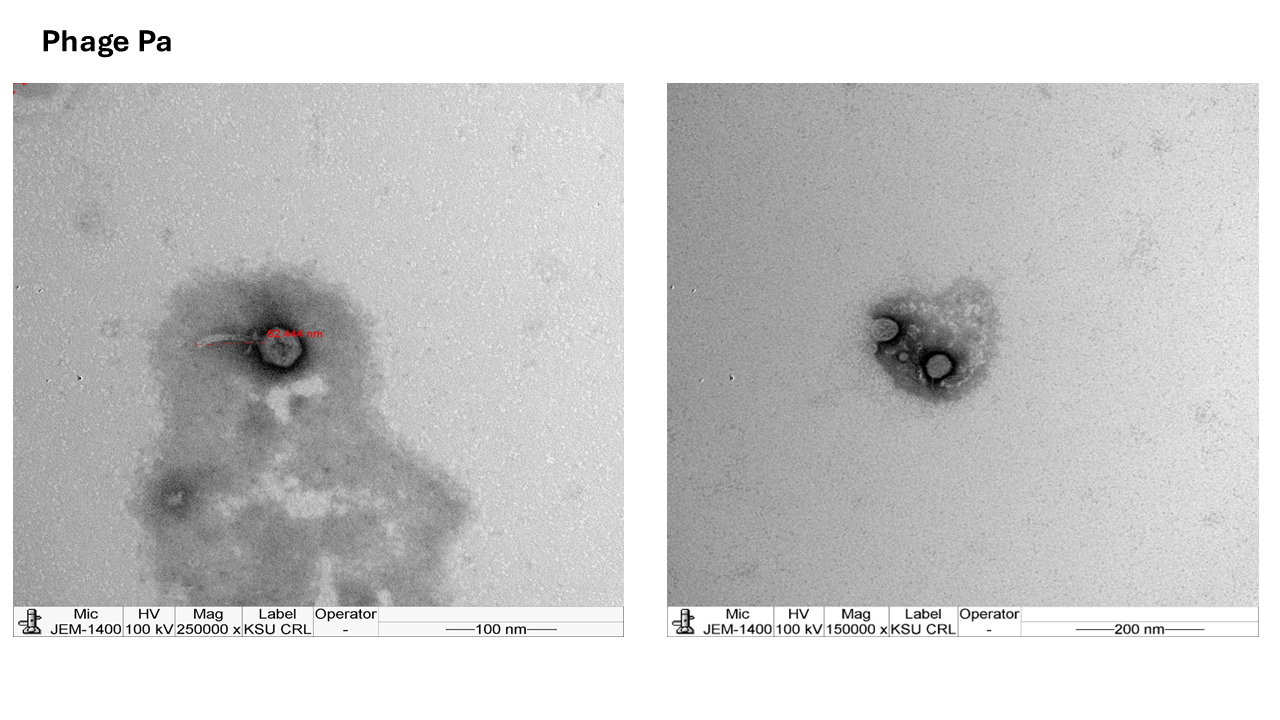

Supplement: Supplementary file 1 [file pharmaceuticals-19-00363-s001.zip › pharmaceuticals-4126957-supplementary File S1/Phage Pa/Figure S18. TEM-image-03-Phage Pa.TIF]

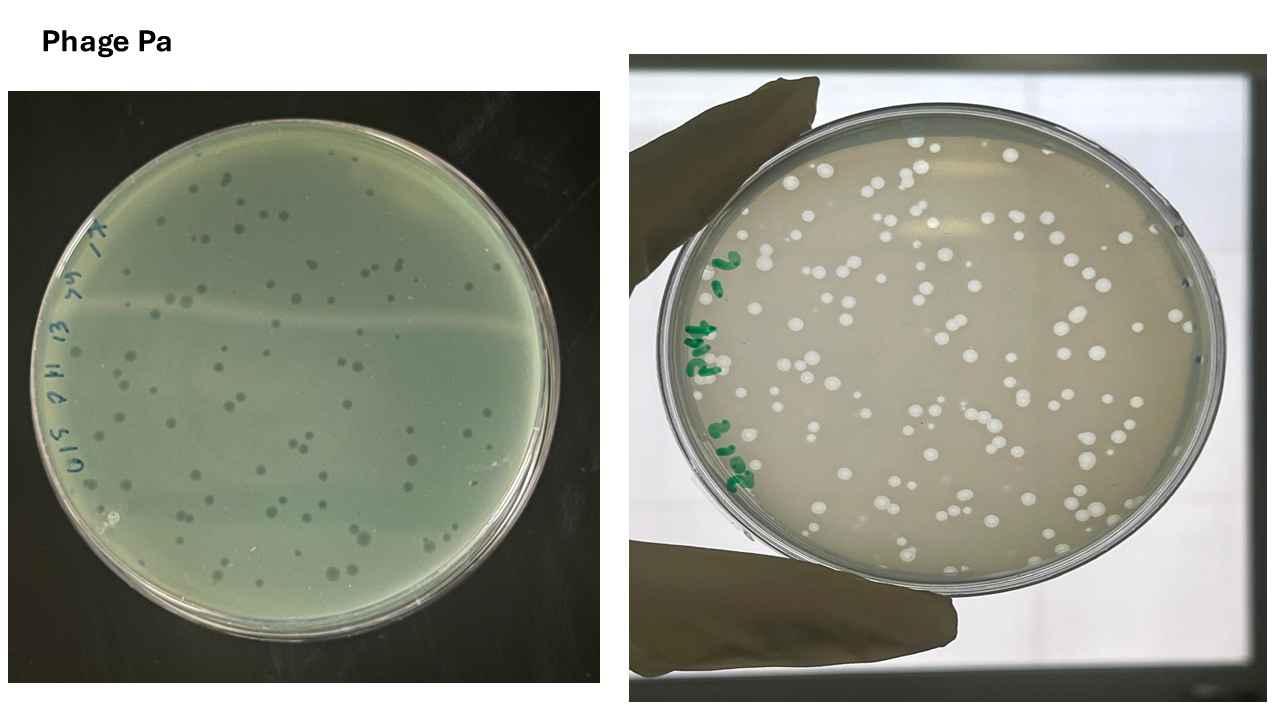

Supplement: Supplementary file 1 [file pharmaceuticals-19-00363-s001.zip › pharmaceuticals-4126957-supplementary File S1/Phage Pa/Figure S5. Phage Pa-Plaque Assay-01.TIF]

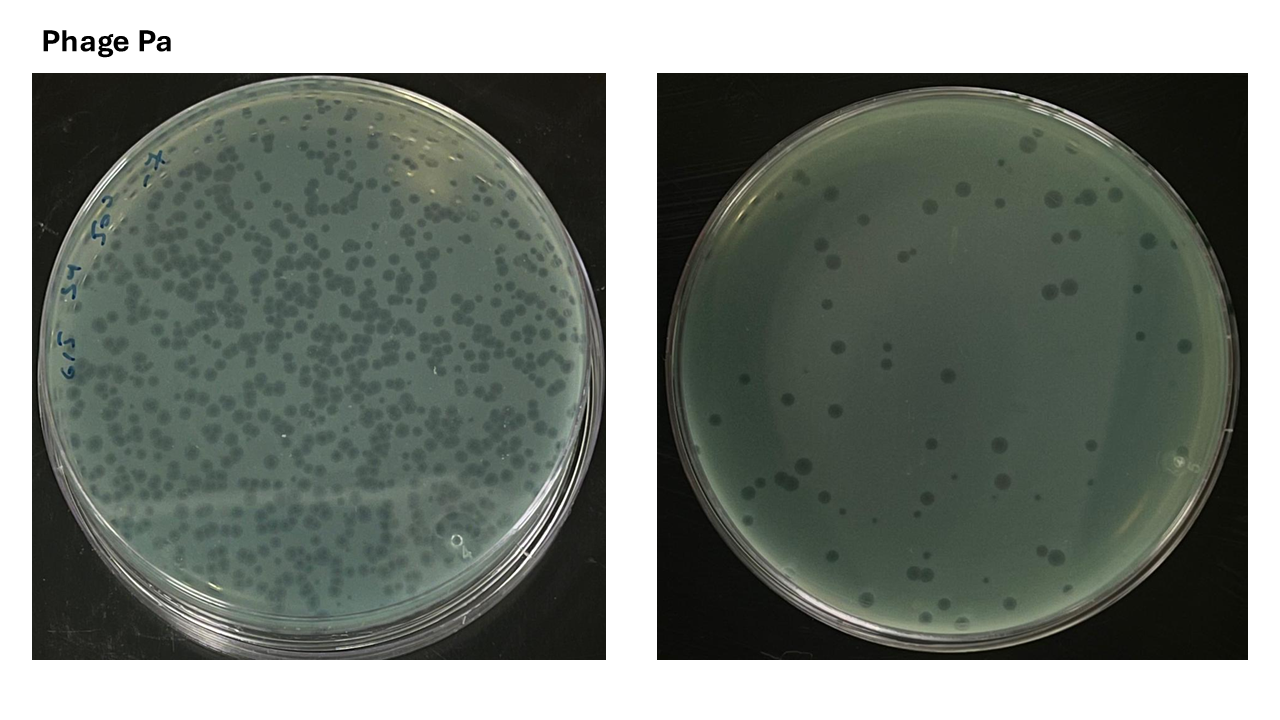

Supplement: Supplementary file 1 [file pharmaceuticals-19-00363-s001.zip › pharmaceuticals-4126957-supplementary File S1/Phage Pa/Figure S6. Phage Pa-Plaque Assay-02.TIF]

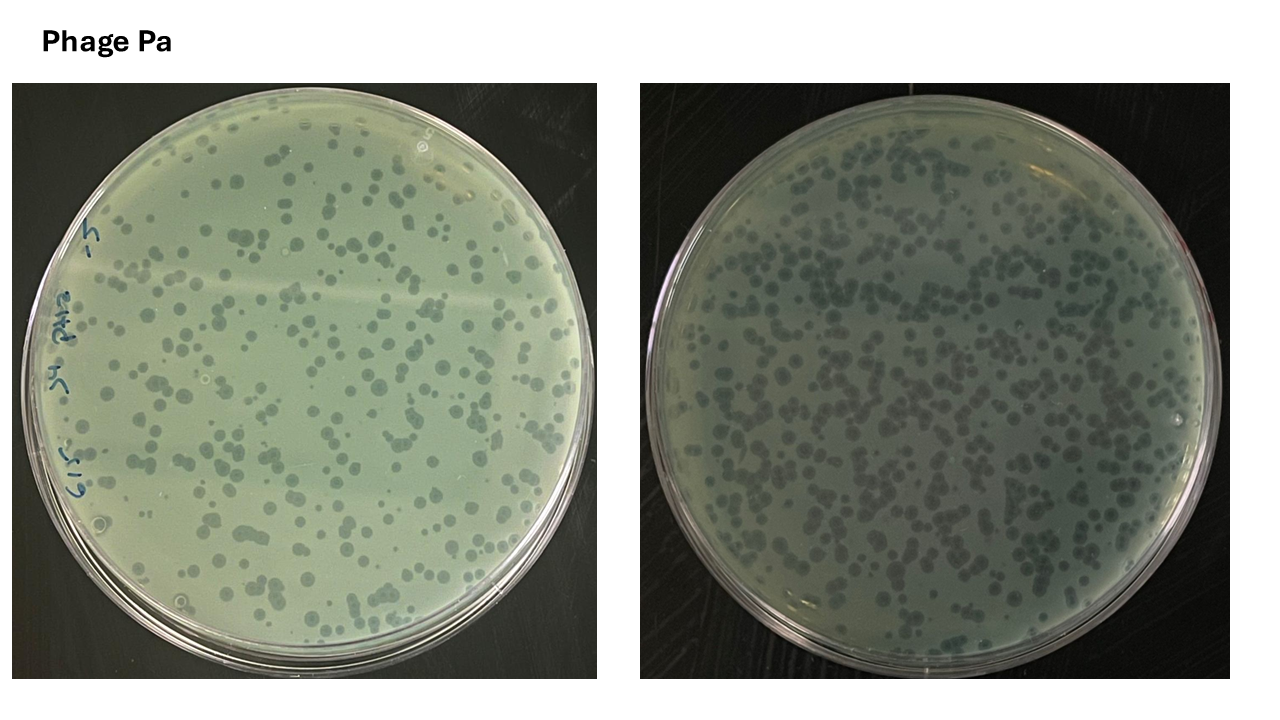

Supplement: Supplementary file 1 [file pharmaceuticals-19-00363-s001.zip › pharmaceuticals-4126957-supplementary File S1/Phage Pa/Figure S7. Phage Pa-Plaque Assay-03.TIF]
